# Bioinspired Microreactor for Iodide Adsorption and Photooxidation Recovery

**DOI:** 10.1002/advs.202518202

**Published:** 2026-02-25

**Authors:** Xuewen Cao, Xuefeng Tian, Jun Zhang, Jinjiao Pan, Rui Huang, Xinfeng Du, Yihui Yuan, Ning Wang, Hui Wang

**Affiliations:** ^1^ School of Marine Sciences State Key Laboratory of Marine Resource Utilization in South China Sea Hainan University Haikou P. R. China

**Keywords:** bioinspired microreactor, brine, iodide ion, photooxidation, recovery capacity

## Abstract

Iodine, an indispensable element for both industry and biology, suffers from scarcity in the earth's crust and inefficiency in conventional recovery technologies. Herein, inspired by the iodide oxidation pathway in thyroid follicular lumens, we designed a bioinspired micro‐ionic‐reactor based on a porous organic polymer (MIR‐POP) that integrates iodide capture with in situ photooxidative conversion. The cationic framework of MIR‐POP enables ultrafast electrostatic enrichment of iodide (I^−^) ions within confined pores, where subsequent light irradiation drives their transformation into molecular iodine and polyiodide species. This bioinspired strategy outperforms adsorption‐based materials and achieves a record uptake capacity of 853.06 mg g^−1^. Moreover, MIR‐POP exhibits remarkable selectivity toward competing anions and delivers high recovery efficiencies of 93.8% in simulated mining wastewater and 85.8% in natural brine, highlighting its promising potential in complex environments. This bioinspired microreactor platform opens a new avenue for selective I^−^ ions recovery and in situ conversion, advancing both environmental remediation and strategic iodine resource recovery.

## Graphical abstract

A bioinspired micro‐ionic‐reactor based on porous organic polymer (MIR‐POP) integrates rapid iodide capture with in situ photooxidative conversion, achieving record uptake capacity and high uptake efficiencies in simulated mining wastewater and natural brine. This bioinspired microreactor strategy offers a new avenue for sustainable iodine recovery and environmental remediation.

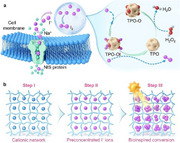

## 1 Introduction

Iodine, a critical industrial and biologically essential trace element, exists in extremely low abundance in the earth's crust with an average content of only 0.46 ppm, making it one of the rarest stable halogens [1, 2, 3, 4]. Current global iodine supply heavily relies on nitrate ores, natural iodine‐rich brines, and oilfield‐produced waters [5, 6, 7]. According to the United States Geological Survey data, global iodine production was approximately 30 000 metric tons, with nitrate ores contributing around 63% and iodine‐rich brines about 30% [8]. Despite the substantial terrestrial iodine reserves, significant challenges persist in its extraction. The industrial process typically involves strong acid leaching, oxidative conversion, air stripping, and elution regeneration, encompassing multiple chemical reagents and engineering steps [9, 10, 11]. These workflows not only demand high energy input but also generate large volumes of iodine‐containing acidic wastewater, resulting in severe equipment corrosion and ecological risks due to the toxicity and bioaccumulative potential of iodine species. Iodide ion (I^−^) is the predominant dissolved iodine species and is typically oxidized to molecular iodine prior to recovery through separation and purification, a pathway that entails high reagent consumption, operational complexity, and secondary waste generation. Therefore, developing advanced systems capable of efficiently and readily recovering iodide is essential for mitigating iodine contamination and enabling sustainable iodine resource extraction.

Extensive efforts have been devoted to the design of functional materials for the efficient removal of I^−^ ions from aqueous media. Various adsorbents, including metal‐organic frameworks (MOFs) [12], covalent organic frameworks (COFs) [13], porous aromatic frameworks [14], layered double hydroxides (LDHs) [15], and metallic oxide composites [16], have been explored for their I^−^ ion adsorption capabilities. Common strategies for achieving effective I^−^ ion capture include (i) electrostatic ion exchange, where I^−^ ions are exchanged with exchangeable anionic groups embedded in porous organic polymers or interlayer anions in LDHs [17, 18]; (ii) chemisorption interactions, where high‐affinity metal sites such as Ag and Cu centers establish strong and specific coordination with I^−^ ions [19, 20, 21]; (iii) structural confinement within porous frameworks, where tuned pore size and local charge environments facilitate the stabilization and enrichment of I^−^ ions [22, 23]. Although these approaches have significantly advanced the design of functional adsorbents and demonstrated the potential for I^−^ ion capture from aqueous conditions, adsorption‐based methods remain fundamentally constrained by thermodynamic equilibria and adsorption kinetics, which impose intrinsic limits on uptake capacity, rate, and selectivity. In addition, the oxidation of I^−^ ions to molecular iodine represents a pivotal step in industrial iodine production, as the high solubility and strong hydration of I^−^ ions in aqueous media make direct separation challenging. The liberation of I^−^ ions is industrially accomplished by oxidizing the acidified leaching solution using chlorine gas, which suffers from severe drawbacks such as strong corrosiveness, over‐oxidation side reactions, and the generation of chlorine‐containing secondary wastes [24]. Therefore, developing integrated strategies that combine I^−^ ion enrichment with in situ conversion into recoverable iodine is highly desirable, as this dual‐function approach could improve I^−^ ion uptake efficiency and reduce environmental risks.

**FIGURE 1:**
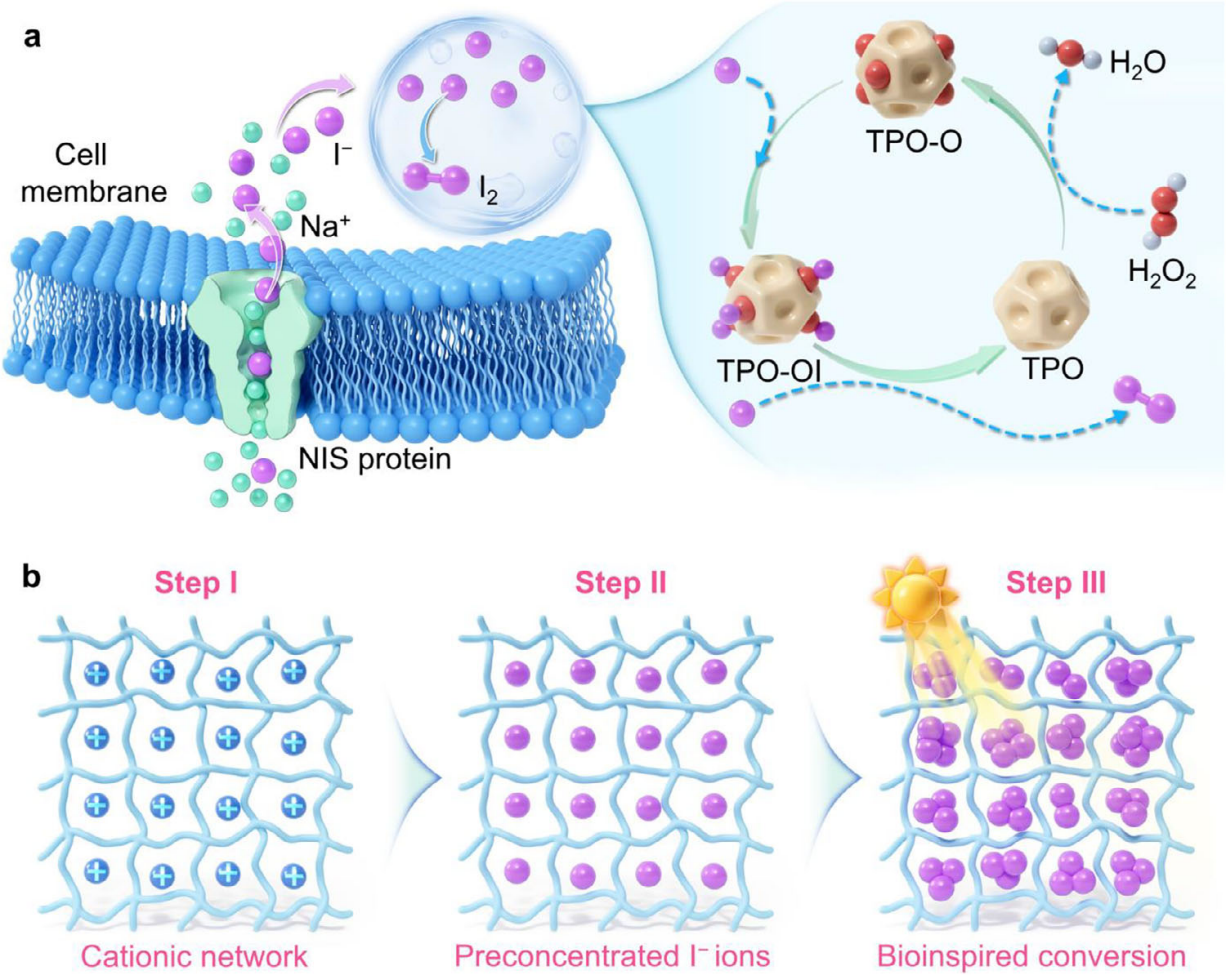
Schematic illustration for the design of a bioinspired ionic microreactor. (a) Schematic illustration of the natural I^−^ ions uptake and oxidation pathway in thyroid follicular cells. (b) Schematic illustration for the mechanism of the bioinspired microreactor constructed based on the porous organic polymer platform for I^−^ ions recovery. Step I indicates the cationic porous organic polymer framework that can electrostatically attract negatively charged I^−^ ions. Step II indicates that I^−^ ions are preconcentrated within the confined microenvironment. Step III indicates that upon light irradiation, the preconcentrated I^−^ ions undergo oxidative conversion into polyiodide species, yielding a light‐activated microreactor that mimics the enzymatic oxidation process in thyroid follicular lumens.

Given the persistent challenges of achieving efficient enrichment‐oxidation coupling in synthetic systems, nature provides a compelling blueprint. Biological processes have evolved highly orchestrated pathways that achieve both selective I^−^ ion uptake and precisely regulated oxidative conversion, thereby ensuring efficient utilization and stable retention of iodine [25, 26, 27]. A paradigmatic example is the I^−^ ion transport and activation pathway in human thyroid follicular lumens (Figure 1a). In this process, extracellular I^−^ ions are actively transported into follicular epithelial cells via the sodium/iodide symporter (NIS) and subsequently translocated across the apical membrane into the follicular lumen [28, 29]. Within this confined microreactor‐like environment, I^−^ ions are rapidly oxidized to reactive iodine species (e.g., I_2_) by thyroid peroxidase (TPO) in the presence of H_2_O_2_ [30, 31]. This biological process, involving localized accumulation of I^−^ ions and subsequent confined oxidative conversion, ensures precise iodine capture while offering a conceptual framework for constructing synthetic microreactor systems capable of efficient iodide recognition and chemical transformation. However, the transport‐oxidation process in follicular lumens has yet to be explored in the context of iodine pollution remediation or iodine resource recovery, underscoring the promise of bioinspired microreactor systems that combine I^−^ ion recognition with confined oxidative ability to achieve simultaneous capture, conversion, and stabilization of iodide species.

In this study, inspired by the iodide oxidation pathway in thyroid follicular lumens, we constructed a bioinspired micro‐ionic‐reactor based on the porous organic polymer (MIR‐POP) platform, which integrates ionic functionalities, photoactive moieties, and fine‐tuned pore environments within the network to ensure intimate coupling between the local chemical microenvironment and photoactivation (Figure 1b). Under light irradiation, the MIR‐POP system enables ultrafast I^−^ ions enrichment followed by in situ photooxidation to molecular iodine and polyiodide (I_3_^−^ and I_5_^−^), enabling a groundbreaking uptake capacity of 853.06 mg g^−1^. By integrating enrichment and transformation within the porous networks, this strategy addresses the limitations of conventional adsorption‐based materials, including low capacity and slow adsorption rate, while dispensing with external oxidants and multistep operations in industrial iodine recovery. This bioinspired microreactor strategy provides valuable guidance for future iodine pollution remediation and iodine resource recovery.

## 2 Results and Discussion

Pristine porous organic polymer (POP) was synthesized via a quaternization reaction between 2,4,6‐tris(4‐(bromomethyl)‐phenyl)‐1,3,5‐triazine and 1,3,5‐tri(1H‐imidazol‐1‐yl)benzene at 80°C for 24 h (Figure S1). Considering the environmental concerns associated with the high toxicity of Br^−^, the resulting polymer was immersed in an aqueous NaCl solution to facilitate anion exchange, thereby affording the bioinspired micro‐ionic‐reactor porous organic polymer (MIR‐POP) with improved environmental compatibility. The powder X‐ray diffraction (PXRD) pattern of MIR‐POP exhibits a broad peak at 22°, showing an amorphous character of MIR‐POP similar to that of the previously reported POPs (Figure S2) [32]. The solid‐state ^13^C nuclear magnetic resonance (NMR) spectrum exhibits characteristic peaks at approximately 168 and 134–121 ppm, which can be assigned to the aromatic carbons of the triazine rings, benzene rings, and imidazole rings, respectively (Figure S3). Additionally, the distinct peak at 52 ppm is attributed to the methylene (–CH_2_−) linkers bridging the triazine core with the imidazolium groups, thereby confirming the successful construction of the MIR‐POP framework. For the Fourier‐transform infrared (FT‐IR) spectrum analysis, the characteristic absorption peaks of ─CH_2_− and C–N^+^ at 1417 and 1091 cm^−1^, respectively, are clearly observed, which are consistent with the results in the solid‐state ^13^C NMR spectrum, further validating the successful quaternization and structural integrity of MIR‐POP (Figure S4). The presence of C‐N^+^ moieties acts as positively charged functional sites for the binding of I^−^ ions. The scanning electron microscope (SEM) image reveals that MIR‐POP features an interconnected, sponge‐like porous network composed of twisted fibrillar frameworks, which provides a high surface area and abundant open channels to facilitate rapid ion diffusion and efficient mass transport, thereby enabling effective preconcentration of I^−^ ions (Figure S5). The energy‐dispersive spectroscopy (EDS) mapping confirms the successful anion exchange, as evidenced by the uniform and dense distribution of Cl element through the surface of MIR‐POP (Figure S6). The anion exchange from Br^−^ to Cl^−^ was further confirmed by X‐ray photoelectron spectroscopy (XPS). The disappearance of Br 3*p* and Br 3*d* peaks together with the emergence of the Cl 2*p* peak clearly demonstrates the successful anion exchange from Br^−^ to Cl^−^ in the MIR‐POP (Figure S7). The N_2_ adsorption‐desorption analysis reveals that MIR‐POP possesses a Brunauer–Emmett–Teller (BET) surface area of 71.9 m^2^ g^−1^, further proving its abundant porous structure (Figure S8). The pore size distribution indicates the presence of mesopores in the range of 2–50 nm, which provides open diffusion channels for I^−^ ions and improves the accessibility of the internal network (Figure S9). In addition, smaller pores around 0.8‐1.0 nm distributed within the internal network serve as confined domains that enable the preconcentration of I^−^ ions, thereby creating reaction chambers for the photoinduced transformation and providing favorable spatial accommodation for the polyiodide species (Figure S10) [33].

**FIGURE 2:**
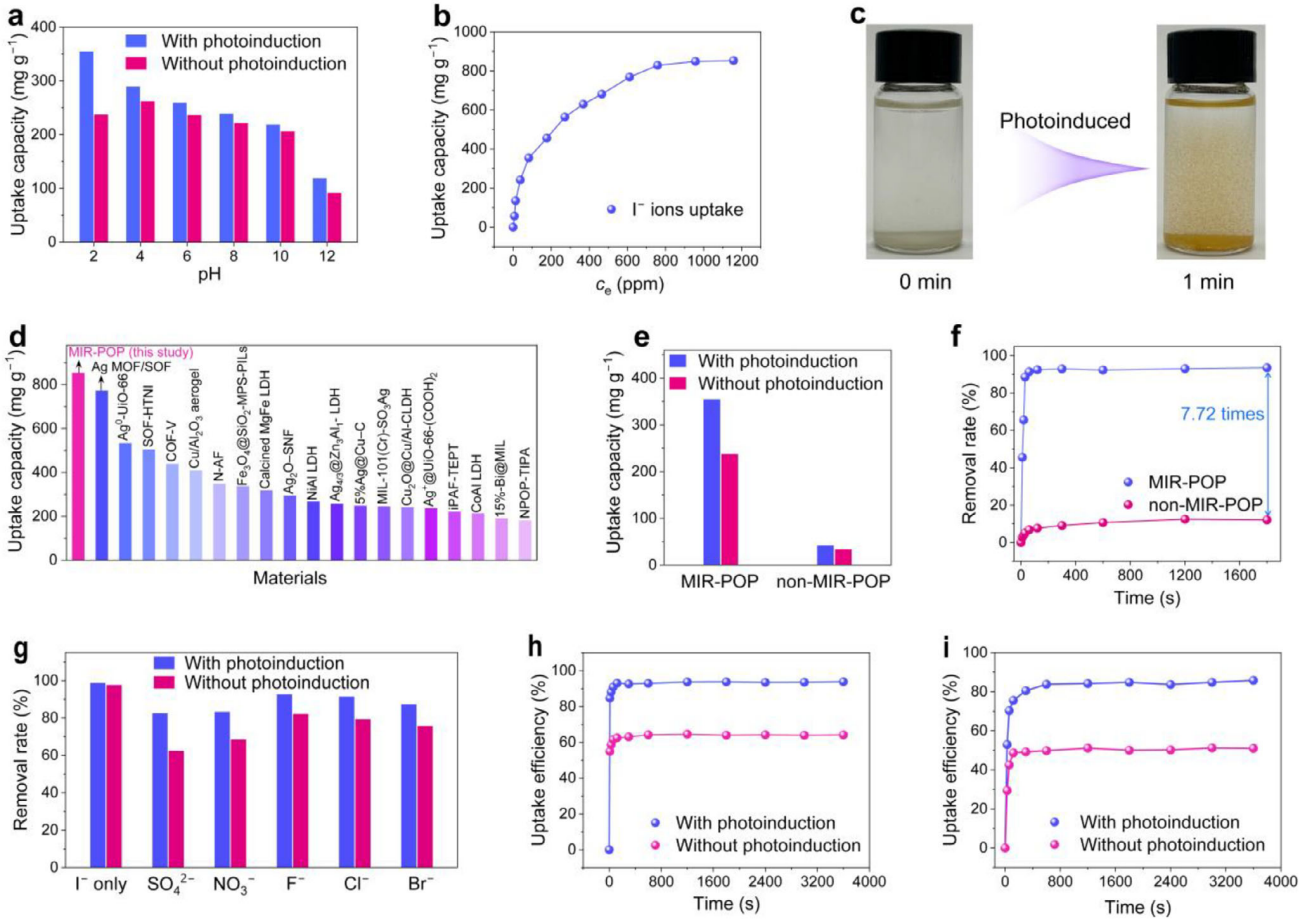
I^−^ ions recovery performance. (a) I^−^ ions uptake capacity of MIR‐POP under different pH values with and without photoinduced conditions at an initial iodide concentration of 100 ppm. (b) I^−^ ions uptake isotherms of MIR‐POP at pH 2 with photoinduction. (c) Color change of MIR‐POP after 1 min exposure to 1200 ppm iodide solution under photoinduced conditions. (d) Comparison of I^−^ ions uptake capacities of available representative materials and MIR‐POP. (e) I^−^ ions uptake capacities of MIR‐POP and non‐MIR‐POP at an initial iodide concentration of 100 ppm and pH 2 with and without photoinduced conditions. (f) The removal kinetics of MIR‐POP and non‐MIR‐POP for I^−^ ions with an initial concentration of 100 ppm at pH 2 with photoinduction. (g) Effect of 50 equivalents of competing anions on the removal of I^−^ ions from aqueous solutions. (h) Uptake kinetics of MIR‐POP for I^−^ ions in simulated mining wastewater at pH 2. (i) Uptake kinetics of MIR‐POP in natural brine at pH 2.

The adsorption performance of MIR‐POP for I^−^ ions was investigated through batch experiments conducted under both dark and photoinduced conditions. Initially, the influence of pH on the I^−^ ions uptake performance of MIR‐POP was examined. Light irradiation enhances the uptake capacity of MIR‐POP across the entire pH range, with the most pronounced effect observed under acidic conditions (Figure 2a; Figure S11). At pH 2, the uptake capacity under photoinduced conditions is 1.49 times higher than that in the dark, highlighting the critical role of photoactivation and acidic conditions in promoting I^−^ ions capture. Considering the excellent I^−^ ion capture of MIR‐POP in acidic media, its zeta potential was further measured to evaluate the protonation state of the network. MIR‐POP maintains high positive zeta potential across pH 2–6, attributed to the positively charged imidazolium moieties, thereby enabling strong electrostatic attraction toward I^−^ ions under acidic environment (Figure S12). Furthermore, the acidic environment thermodynamically favors the oxidation of I^−^ ions to I_2_ under light irradiation [34]. The effect of adsorbent dosage on the I^−^ ions uptake performance was investigated, and the results confirm that the uptake capacity of I^−^ ions increases from 93.60 to 354.36 mg g^−1^ as the adsorbent amount decreases under photoinduced conditions (Figure S13). To determine the maximum uptake capacity of MIR‐POP toward I^−^ ions, adsorption isotherm experiments were performed over a range of iodide concentrations under both photoinduced and without photoinduced conditions. Without photoactivation, MIR‐POP exhibits an adsorption capacity of 556.60 mg g^−1^ and a Langmuir‐fitted theoretical maximum capacity of 612.62 mg g^−1^ for I^−^ ions, with no observable change in the color of MIR‐POP (Figures S14 and S15 and Table S1). Upon photoinduced treatment, the uptake capacity increased markedly to 853.06 mg g^−1^, accompanied by a rapid color change of the material, which highlights the pivotal role of photoinduced conditions in enhancing I^−^ ions extraction efficiency and suggests the possible oxidation of iodide species (Figure 2b,c). Comparison with other materials, including MOF material Ag^0^‐UiO‐66‐(OH)_2_ (531.98 mg g^−1^) [35], the COF material COF‐V (437.80 mg g^−1^) [13], the LDHs material calcined MgFe LDH (317.50 mg g^−1^) [15], and the metallic oxide composites Cu/Al_2_O_3_ aerogel (407.60 mg g^−1^) [36], MIR‐POP exhibits a significantly higher uptake capacity under photoactivation, demonstrating the effectiveness of the bioinspired microreactor strategy based on a cationic polymer network in achieving high capacity I^−^ ions uptake (Figure 2d; Table S2).

To elucidate the role of ionic exchange sites in MIR‐POP for I^−^ ions uptake, a triazine‐based polymer without ion‐exchangeable sites (non‐MIR‐POP) was synthesized via a Schiff base reaction between the 4,4“,4”‐(1,3,5‐Triazine‐2,4,6‐triyl)trianiline and terephthalaldehyde at 120°C for 72 h (Figure S16). The peak at 1625 cm^−1^ in the FT‐IR spectrum is assigned to the C═N stretching vibration of non‐MIR‐POP, confirming the successful synthesis of non‐MIR‐POP (Figure S17). Under light irradiation, non‐MIR‐POP only exhibits the capacity of physical adsorption for I^−^ ions and shows no significant color change, with an uptake capacity of 41.88 mg g^−1^, which is markedly lower than that of MIR‐POP (Figure 2e; Figure S18). This result reveals that the photoactive triazine units alone are insufficient to achieve efficient I^−^ ions recovery and transformation. At pH 2 with an initial iodide concentration of 100 ppm, non‐MIR‐POP exhibits only 5.24% removal at 30 s and eventually reaches merely 12.12% at equilibrium. In sharp contrast, MIR‐POP rapidly removes 88.6% of I^−^ ions within 30 s, ultimately achieving a removal efficiency of 93.6%, which is approximately 7.72 times higher than that of non‐MIR‐POP (Figure 2f). This pronounced disparity highlights that spatial preconcentration of I^−^ ions at ionic exchange sites is indispensable not only for facilitating rapid uptake but also as a prerequisite for subsequent photoinduced oxidation, thereby validating the effectiveness of the bioinspired microreactor strategy in which localized I^−^ ions preconcentration initiates a thyroid‐like oxidative conversion pathway for efficient I^−^ ions uptake under light irradiation.

Given the abundance of competing anions in practical aqueous environments, the uptake selectivity of MIR‐POP toward I^−^ ions was systematically assessed in the presence of large excesses of representative coexisting anions, including SO_4_^2−^, NO_3_^−^, F^−^, Cl^−^, and Br^−^ ions. The I^−^ ion removal performance of MIR‐POP in the absence of photoactivation is strongly influenced by both the concentration and the valency of the competing anions (Figure 2g; Figure S19). Notably, multivalent anions such as SO_4_^2−^ impose strong competitive inhibition, reducing the I^−^ ion removal efficiency to 56.1% when the molar ratio of SO_4_^2−^ to I^−^ ions reaches 100:1. In contrast, under photoactivated conditions, the inhibitory effect of multivalent anions is markedly mitigated, highlighting that the synergistic interplay between electrostatic enrichment and photoinduced oxidation substantially enhances the preferential uptake of I^−^ ions, even in the presence of a large excess of competing species. MIR‐POP maintains high removal efficiencies for I^−^ ions up to 92.5% and 88.1% when the molar ratios of coexisting anions to I^−^ ions reach 50:1 and 100:1, respectively. This robust selectivity arises from the light‐triggered oxidation, which minimizes interference from anions that typically impose severe competitive effects in conventional adsorption‐based materials [37]. Reusability is a key property of adsorbents as it directly determines their long‐term applicability and cost efficiency, and thus, the recyclability of MIR‐POP was evaluated through adsorption‐desorption cycles. After five cycles, MIR‐POP maintains a high removal efficiency of 92.1% for I^−^ ions (Figure S20). To further evaluate the structural stability of MIR‐POP over multiple reuse cycles, characterizations were conducted on the cycled material. The FT‐IR spectra remain unchanged after five cycles, with the characteristic peaks of ─CH_2_− and C─N^+^ still evident, indicating that the chemical structure of the framework is preserved throughout reuse (Figure S21). The PXRD pattern reveals that the cycled MIR‐POP retains the broad amorphous feature of the pristine material (Figure S22). N_2_ adsorption‐desorption analysis reveals a BET surface area of 50.8 m^2^ g^−1^ for the cycled MIR‐POP, demonstrating that the porous structure is well maintained after cycled (Figure S23). In addition, the total organic carbon of the supernatant after cycled remains below the detection limit, indicating that MIR‐POP shows no photocatalytic degradation during repeated photooxidation and thus avoids introducing secondary organic contamination into the aqueous media. These results confirm that MIR‐POP still maintains its structural stability during multiple recycles. Such a bioinspired microreactor strategy effectively enhances the selective uptake of I^−^ ions and the recyclability of materials compared with the sole adsorption strategy, offering a promising approach for practical I^−^ ion extraction from complex aqueous environments.

To further evaluate the practical feasibility of this bioinspired I^−^ ion extraction strategy, we investigated its performance in complex natural aqueous systems. Considering that nearly three‐fifths of the world's iodine production is derived from caliche nitrate ore processing, simulated mining wastewater was chosen as a representative test medium [9, 38]. In simulated wastewater, with its performance evaluated across different solid‐to‐liquid ratios, MIR‐POP achieves a superior I^−^ ion uptake efficiency of 93.8% at a solid‐to‐liquid ratio of 1 g L^−1^, which is significantly higher than the 64.1% efficiency observed without photoactivation (Figure 2h; Figure S24). This result reveals the rapid responsiveness and high removal efficiency of MIR‐POP in simulated wastewater. In addition, given that brine constitutes one of the richest natural iodine reservoirs, the iodine uptake efficiency of MIR‐POP was investigated using a natural sample collected from Dabuxun Lake in Qinghai, with an initial iodine concentration of 432.6 ppb. Various solid‐to‐liquid ratios of MIR‐POP were tested, and the results indicate that the uptake efficiency improves with increasing ratio, with the best performance observed at 10 g L^−1^ (Figure S25). As shown in Figure 2i, MIR‐POP displays a markedly enhanced uptake efficiency under photoinduced conditions, achieving an uptake efficiency of 85.8% compared to 51.0% under dark conditions, representing a 68.2% improvement. Compared with conventional I^−^ ion extraction methods, this bioinspired strategy offers faster response, higher uptake efficiency, and more convenient operation under complex ionic environments, highlighting its promise for practical I^−^ ion recovery.

**FIGURE 3:**
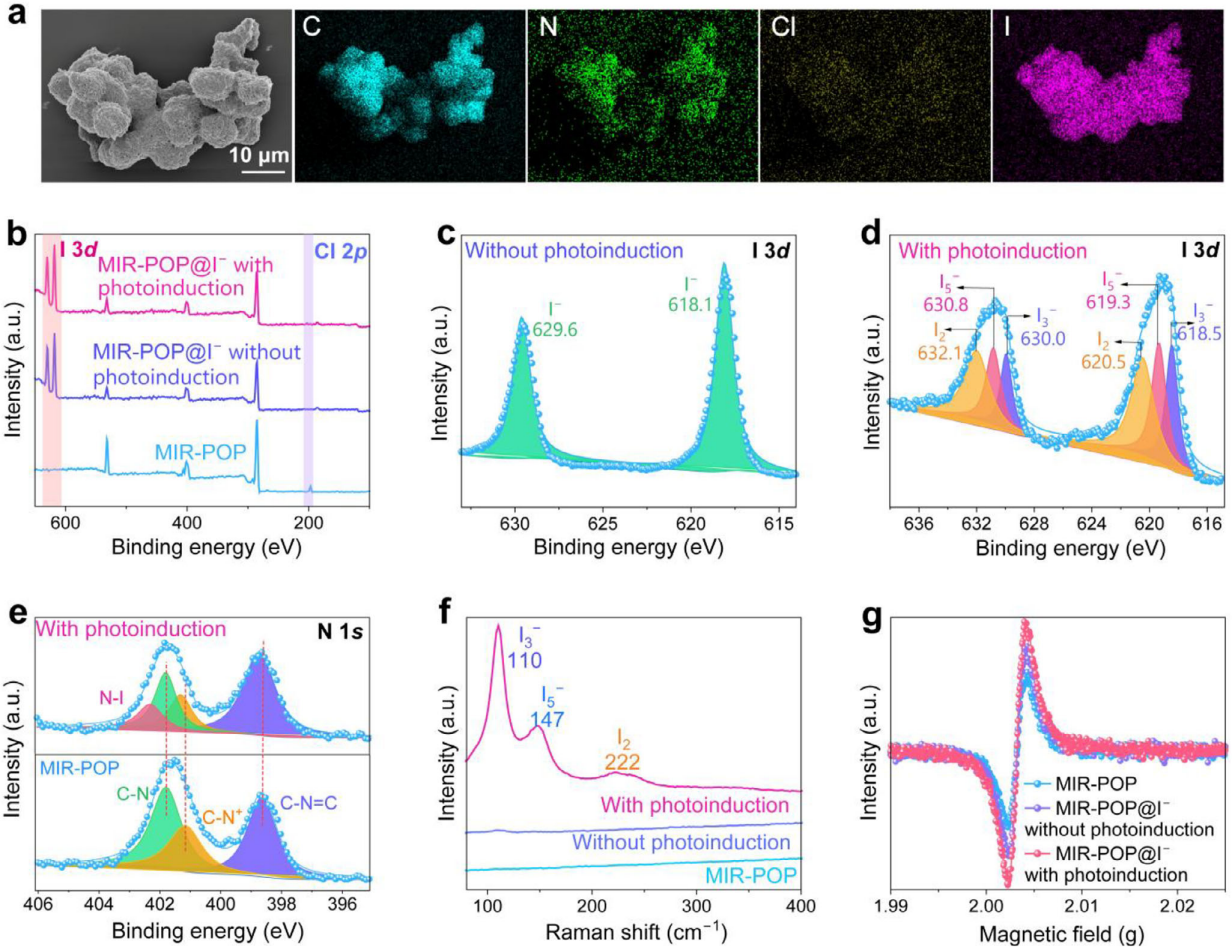
Characterization of MIR‐POP after being used for I^−^ ion recovery. (a) SEM image and corresponding elemental mapping of MIR‐POP after I^−^ ions uptake under photoinduced conditions. (b) XPS spectra of pristine MIR‐POP, MIR‐POP@I^−^ obtained without photoinduction, and MIR‐POP@I^−^ obtained with photoinduction. (c) High‐resolution I 3*d* XPS spectrum of MIR‐POP@I^−^ obtained without photoinduction. (d) High‐resolution I 3*d* XPS spectrum of MIR‐POP@I^−^ obtained with photoinduction. (e) High‐resolution N 1*s* XPS spectra of MIR‐POP before and after I^−^ ions uptake under photoinduction. (f) Raman spectra of pristine MIR‐POP, MIR‐POP@I^−^ obtained without photoinduction, and MIR‐POP@I^−^ obtained with photoinduction. (g) EPR spectra of pristine MIR‐POP, MIR‐POP@I^−^ obtained without photoinduction, and MIR‐POP@I^−^ obtained with photoinduction.

To analyze the mechanism underlying the bioinspired recovery of I^−^ ions by MIR‐POP, systematic characterizations were carried out on pristine MIR‐POP and iodine loaded MIR‐POP (MIR‐POP@I^−^). SEM images of MIR‐POP@I^−^ obtained under light irradiation still retain a sponge‐like porous network structure, demonstrating the structural robustness of MIR‐POP (Figure S26). The corresponding elemental mapping reveals a uniform distribution of iodine on the surface of MIR‐POP, accompanied by a notable decrease in Cl content (Figure 3a). This indicates that the preconcentration of I^−^ ions is mediated by anion exchange with Cl^−^ binding sites, in agreement with the experimental observations. XPS analysis of MIR‐POP after I^−^ ions uptake, both with and without photoinduction, reveals the emergence of characteristic I 3*d* signals accompanied by the disappearance of Cl 2*p* signal, which is consistent with the elemental distribution observed in SEM‐EDS mapping (Figure 3b). As shown in the high‐resolution I 3*d* XPS spectra, distinct differences in the chemical states of iodine species are observed between photoinduced and without photoinduced conditions. For MIR‐POP@I^−^ without photoinduction, two prominent peaks located at 629.6 eV and 618.1 eV are assigned to the I 3*d*_3/2_ and I 3*d*_5/2_ spin‐orbit components of monovalent iodide, respectively, confirming that MIR‐POP mainly acts as an ion‐exchange matrix without any iodide transformation (Figure 3c). In contrast, the iodine species in MIR‐POP@I^−^ under photoinduced conditions are classified as the molecular iodine and polyiodide rather than remaining as adsorbed I^−^ ion without light irradiation. Specifically, the I 3*d* spectrum exhibits characteristic peaks at 630.0 and 618.5 eV, 630.8 and 619.3 eV, and 632.1 and 620.5 eV, which can be attributed to triiodide (I_3_^−^), pentaiodide (I_5_^−^), and molecular iodine (I_2_), respectively (Figure 3d). This photoinduced transformation of I^−^ ions confirms the oxidative recovery mechanism and emphasizes the microreactor‐like functionality of MIR‐POP. Furthermore, the high‐resolution N 1*s* XPS spectra reveal the binding energy shifts of the C = N−C, C−N^+^, and C−N peaks from 398.6 to 398.7 eV, from 401.1 to 401.2 eV and from 401.7 to 401.8 eV upon photoinduced I^−^ ions uptake, respectively, demonstrating the formation of charge‐transfer complexes between the polyiodide species and nitrogen sites on MIR‐POP (Figure 3e) [39]. The appearance of a new peak at 402.3 eV corresponds to N–I interactions, further substantiating the existence of such charge‐transfer complexes and reinforcing the critical role of nitrogen functionalities in mediating photoinduced iodine recovery. To further validate the formation of polyiodide species, Raman spectroscopy was performed on MIR‐POP before and after photoinduced I^−^ ions uptake (Figure 3f). Both the pristine MIR‐POP and the I^−^ ion treated sample without photoinduction show no discernible features in the 100–400 cm^−1^ range, thereby proving that no iodine species are present in either sample. By contrast, MIR‐POP exposure to I^−^ ion solution under light irradiation exhibits three distinct Raman bands at 110, 147, and 222 cm^−1^, which are attributed to the symmetric stretching of I_3_^−^, I_5_^−^, and I_2_, respectively [40]. These characteristic peaks provide direct spectroscopic evidence for the light‐driven oxidative transformation of preconcentrated I^−^ ions into molecular iodine and polyiodide species, in agreement with the XPS results. The presence of charge‐transfer interactions between MIR‐POP and iodine species was further evidenced by electron paramagnetic resonance (EPR) spectroscopy. As shown in Figure 3g, both MIR‐POP@I^−^ samples, with and without photoinduction, exhibit enhanced signal intensity than pristine MIR‐POP, indicating the presence of electron transfer interactions between iodine species and the cationic polymer network. Importantly, the higher signal intensity observed after I^−^ ion uptake under photoinduced conditions suggests the formation of unpaired electrons associated with reactive iodine intermediates, which confirms the participation of photoactivated radical pathways in the oxidative transformation of I^−^ ions within the MIR‐POP network [41, 42, 43].

**FIGURE 4:**
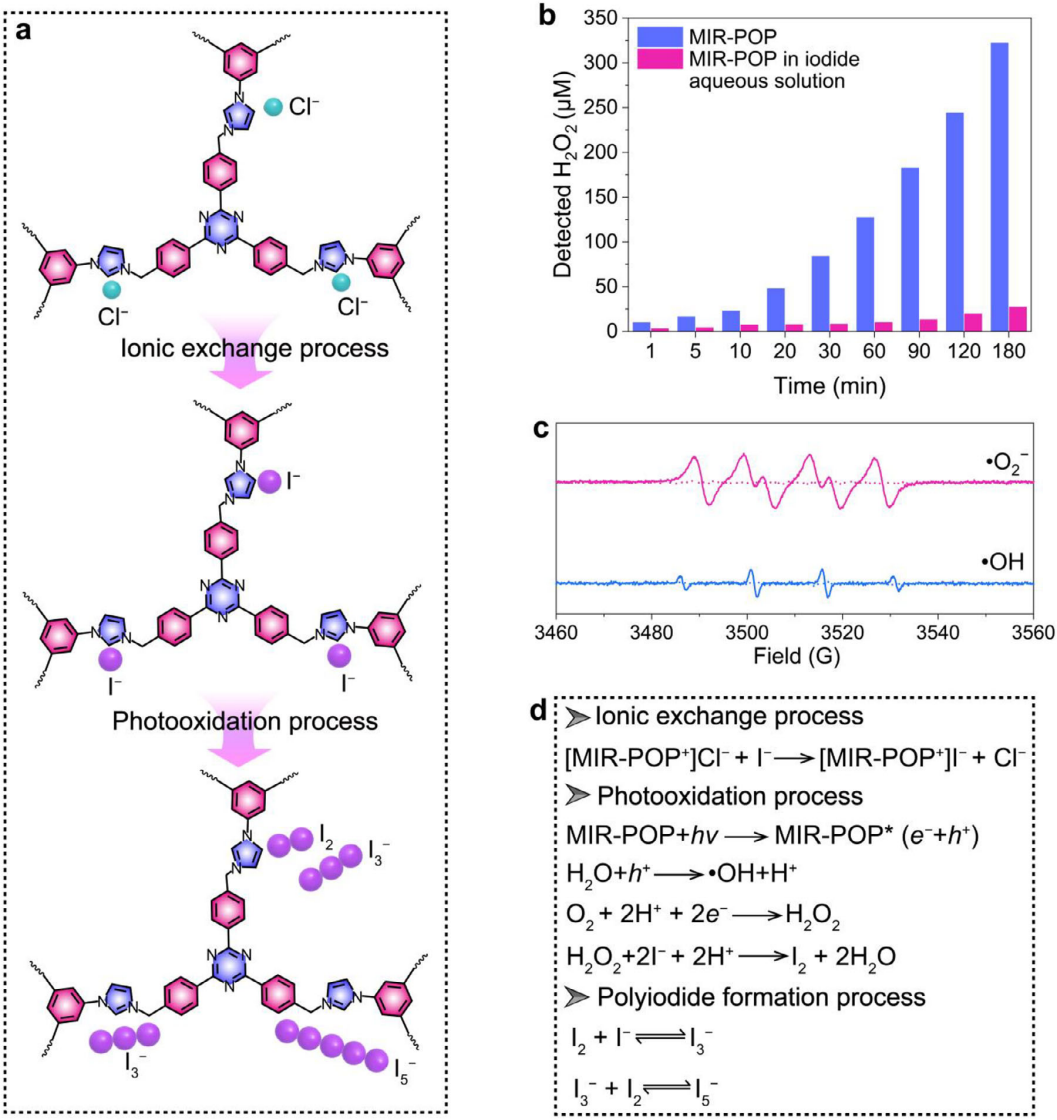
Mechanism for I^−^ ions recovery by MIR‐POP. (a) The ionic exchange process and photooxidation process of MIR‐POP for I^−^ ions recovery. (b) Time‐dependent generation of H_2_O_2_ by MIR‐POP with photoinduction in the absence and presence of I^−^ ions. (c) EPR spectra for DMPO‐·O_2_^−^ and DMPO‐·OH complexes formed under visible light irradiation for MIR‐POP. (d) Proposed reaction pathway for photoinduced I^−^ ions oxidation over MIR‐POP.

Based on the above characterization analyses, the photoinduced transformation pathway of I^−^ ions over MIR‐POP was elucidated. It has been proven that the photoactivation of I^−^ ions proceeds through a sequential ionic exchange and light‐driven activation pathway (Figure 4a). Since H_2_O_2_ functions as the essential participant in thyroid hormone biosynthesis, its generation provides a key indicator of the bioinspired microreactor oxidation. Time‐dependent formation of H_2_O_2_ under light irradiation for MIR‐POP both in the absence and presence of I^−^ ions was quantified using a colorimetric assay (Figure 4b). In the I^−^ ions‐free system, the concentration of H_2_O_2_ increases continuously from 10.02 µm at 1 min to 322.21 µm at 180 min, exhibiting a near‐linear growth trend, indicative of the sustained and efficient H_2_O_2_ generation capability of MIR‐POP under photoinduced conditions. When I^−^ ions are added into the system, the H_2_O_2_ concentration declines rapidly to only 27.56 µm at 180 min, indicating its consumption during the photooxidation process of I^−^ ions. EPR spin‐trapping measurements were employed to identify the radical species formed during the photoactivation of I^−^ ions over MIR‐POP (Figure 4c). No EPR signals were detected for MIR‐POP under dark conditions. Under light irradiation, O_2_^−^ and ·OH were detected and trapped using 3,4‐dihydro‐2,3‐dimethyl‐2H‐pyrrole 1‐oxide (DMPO), confirming the formation of reactive oxygen species. The electron‐accepting triazine rings within MIR‐POP facilitate the oxygen reduction reaction, thereby driving the stepwise two‐electron reduction of O_2_ to H_2_O_2_ under photoexcitation, consistent with the H_2_O_2_ production pathway reported for triazine‐based polymer photocatalysts (Figure 4d) [44]. The triazine‐driven oxygen reduction pathway provides the photooxidative drive essential for initiating the bioinspired microreactor. Subsequently, the generated H_2_O_2_ serves as the primary oxidant for the conversion of I^−^ ions into molecular iodine, which is further stabilized into polyiodide species of I_3_^−^ and I_5_^−^ under excess I^−^ ions conditions, as has been previously confirmed in iodine chemical oscillators [45]. This sequence of redox and polyiodide formation steps effectively couples reactive oxygen species generation with selective I^−^ ions oxidation, where the MIR‐POP network functions as a photoinduced ionic microreactor. Such a mechanism parallels the thyroid peroxidase‐mediated pathway of I^−^ ions oxidation, in which H_2_O_2_ acts as the oxidant to convert I^−^ ions into molecular iodine, thereby achieving the simultaneous efficient capture and transformation of I^−^ ions within an aqueous environment.

## 3 Conclusion

In this work, we have developed a thyroid‐inspired micro‐ionic‐reactor that unifies iodide enrichment and photooxidative conversion into a single porous organic framework. By mimicking the natural follicular lumen pathway, MIR‐POP achieves unprecedented iodide uptake capacity and rapid kinetics without external oxidants, while maintaining high selectivity under strongly competitive ionic conditions. Comprehensive mechanistic analyses reveal that the bioinspired microreactor environment facilitates I^−^ ions preconcentration, rapid H_2_O_2_ generation, and subsequent oxidation through a pathway analogous to enzymatic activation in biological systems. This bioinspired system demonstrates superior uptake capacities compared with state‐of‐the‐art adsorbents and exhibits superior iodine capture ability in simulated industrial wastewater and natural brines, highlighting its promising potential in treating mining wastewater and recycling iodine resources. The light‐driven enrichment‐conversion synergy not only enhances extraction efficiency but also minimizes secondary waste generation, addressing the critical drawbacks of conventional adsorption or chlorine‐based oxidation processes. This thyroid‐like strategy demonstrates the potential of bioinspired microreactors to reconcile efficient resource recovery with environmental sustainability. The concept of confined enrichment‐oxidation coupling provides a versatile framework for designing next‐generation functional materials capable of addressing broader challenges in selective ion recovery, pollution control, and sustainable element utilization.

## Conflicts of Interest

The authors declare no conflicts of interest.

## Supporting information




**Supporting File**: advs74375‐sup‐0001‐SuppMat.docx.

## Data Availability

The data that support the findings of this study are available from the corresponding author upon reasonable request.
