# Supplementary material for: Bioinspired Microreactor for Iodide Adsorption and Photooxidation Recovery
Source: Adv Sci (Weinh). 2026 Feb 25;13(23):e18202. doi: 10.1002/advs.202518202 (PMC13104070; doi:10.1002/advs.202518202)
Supplement: Supplementary file 1 — Supporting File: advs74375‐sup‐0001‐SuppMat.docx. [file ADVS-13-e18202-s001.docx]

Supporting Information

**Bioinspired Microreactor for Iodide Adsorption and Photooxidation Recovery**

*Xuewen Cao, Xuefeng Tian, Jun Zhang, Hui Wang,* Jinjiao Pan, Rui Huang, Xinfeng Du, Ning Wang,* Yihui Yuan**

School of Marine Sciences, State Key Laboratory of Marine Resource Utilization in South China Sea

Hainan University

Haikou 570228, P. R. China

*Corresponding authors

E-mail: huiw0318@163.com (H. Wang), wangn02@foxmail.com (N. Wang), yuanyh@hainanu.edu.cn (Y. Yuan)

Table of Contents

[**Experimental Procedures** 3](#_Toc204212820)

[**Supporting Figures and Tables** 7](#_Toc204212821)

[**References** 35](#_Toc204212822)

Experimental Procedures

**Materials**

Chemical reagents and raw materials were purchased from commercial sources and used as received without further treatment. 2,4,6-tris(4-(bromomethyl)- phenyl)-1,3,5-triazine, 1,3,5-tri(1H-imidazol-1-yl)benzene, 4,4’,4”-(1,3,5-Triazine-2,4,6-triyl) trianiline, and terephthalaldehyde were purchased from Jilin Chinese Academy of Sciences-Yanshen Technology Co., Ltd.

**Methods**

Preparation of MIR-POP

A mixture of 2,4,6-tris(4-(bromomethyl)phenyl)-1,3,5-triazine (0.5 mmol, 294 mg) and 1,3,5-tri(1H-imidazol-1-yl)benzene (0.5 mmol, 138 mg) was dissolved in 50 mL of DMF and stirred at 80 °C for 24 h under a nitrogen atmosphere. The resultant powder was collected by filtration and washed with DMF (3 × 50 mL) and MeOH (3 × 50 mL). After immersion in 1 M NaCl aqueous solution (100 mL × 3) for 24 h, the resulting powder was washed with deionized water (100 mL × 3) for 24 h and vacuum-dried at 60 °C for 12 h.

Preparation of non-MIR-POP

A mixture of 4,4',4''-(1,3,5-triazine-2,4,6-triyl)trianiline (0.08 mmol, 28.4 mg) and terephthalaldehyde (0.12 mmol, 16.1 mg) was suspended in a binary solvent system composed of *n*-butanol (1.2 mL) and *o*-dichlorobenzene (0.8 mL), followed by the addition of an aqueous acetic acid solution (6 M, 0.2 mL). After three consecutive freeze-pump-thaw cycles to ensure complete deaeration, the Pyrex reaction tube (10 mL) was flame-sealed under vacuum and heated at 120 °C for 72 h. The resulting precipitate was isolated by filtration, extensively washed with DMF and EtOH to remove unreacted monomers and oligomers, and finally dried under dynamic vacuum at 80 °C for 12 h.

**Characterization**

Fourier transform infrared spectra were measured on a spectrometer (FT-IR, LR 64912C, Perkin-Elmer, USA). Raman spectra were collected using a spectrometer (Raman, HR800, Horiba Jobin Yvon, France). Cross-polarization magic angle spinning ^13^C NMR spectroscopy was conducted with an instrument (^13^C NMR, Bruker BioSpin GmbH, Germany). X-ray diﬀraction pattern of the material was performed by using Rigaku SmartLab diffractometer equipped with a Cu Kα radiation source (XRD, SmartLab 9kW, Rigaku, Japan). N_2_ adsorption-desorption isotherms were collected at 77 K using an automatic analyzer after the samples had been degassed at 100 °C under vacuum for 12 h (ASAP 2460, Mike, USA). The pore size distribution of MIR-POP in the mesoporous region was calculated by the Barrett–Joyner–Halenda method, while the pore size distribution of MIR-POP in the microporous region was calculated by the Horvath–Kawazoe model. Elemental electron binding energy was obtained on an X-ray photoelectron spectrometer (XPS, ESCALAB 250Xi, Thermo, USA). The microscopic morphology and structure of materials were obtained on a field emission scanning electron microscope (SEM, S-4800, HITACHI, Japan). Electron paramagnetic resonance spectra were analyzed with a spectrometer (EPR, A300-10/12, Bruker, Germany). The pH values were detected via a pH meter (F2, Mettler Toledo, Germany). Zeta potential measurement was performed using a zeta potential analyzer (Zetasizer Nano ZSE, Malvern Panalytical, UK). The concentration of I^−^ ions was studied on an inductively coupled plasma mass spectrometer (ICP-MS, iCAP RQ, Thermo Fisher, USA), and the ion concentrations were calculated from a suitable calibration curve (*R*^2^ ≥ 0.9999**). The concentration of total organic carbon in the supernatant was determined on an analyzer (TOC, NexION 5000G, Elementar/precisION, USA).

**Batch adsorption experiments**

All adsorption experiments were conducted under batch conditions. To assess the effect of light irradiation on I^−^ ions adsorption performance, a 300 W Xe lamp with a light intensity of 1 kW·m^−2^ was employed as the simulated sunlight, while the control adsorption experiments were carried out in a dark environment without photoinduced condition. The following experiments were performed under both light-irradiated and dark conditions unless otherwise specified.

The adsorption performance of MIR-POP toward I^−^ ions was evaluated across a pH range of 2–12. The adsorbent with a dry weight of 5 mg was immersed in 100 mL of 100 ppm iodide solution with moderate stirring at 25 °C for 30 min. The residual I^−^ ions concentration was measured using ICP-MS and the pH-dependent adsorption capacity was determined by the following equation:

| 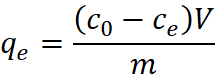 | (1) |
| --- | --- |

where *q*_e_ (mg·g^−1^) is the adsorption capacity of I^−^ ions, *c*_0_ (mg·L^−1^) is the concentration of I^−^ under initial condition and *c*_e_ (mg·L^−1^) is the concentration of I^−^ under equilibrium condition. *V* (mL) is the volume of the solution, and *m* (mg) is the amount of adsorbent.

To investigate the effect of adsorbent amount on the uptake capacity of MIR-POP for I^−^ ions, the adsorbent amount of MIR-POP was systematically varied from 5 mg to 100 mg in 100 mL of iodide solution with an initial concentration of 100 ppm at pH 2. To investigate the adsorption capacity of MIR-POP for I^−^ ions, isothermal experiments were performed. The initial concentrations of I^−^ ions were set as 10 ppm, 20 ppm, 50 ppm, 100 ppm, 200 ppm, 300 ppm, 400 ppm, 500 ppm, 650 ppm, 800 ppm, 1000 ppm, and 1200 ppm. pH value was adjusted to 2 and the adsorption time was set to 30 min. To obtain the corresponding adsorption parameters, the equilibrium data under dark condition were fitted using Langmuir and Freundlich adsorption isotherm equations:

| 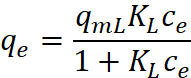 | (2) |
| --- | --- |
| 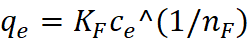 | (3) |

where *K*_L_ (L mg^−1^) and *K*_F_ (mg g^−1^) (L mg^−1^)^1/n^ are defined as the constants of the Langmuir and the Freundlich model. *q*_mL_ (mg g^−1^) denotes the Langmuir maximum adsorption capacity. *n*_F_ (g L^−1^) indicates the adsorption affinity. *q*_e_ and *c*_e_ have the same meaning as Equation (1).

Kinetics tests on I^−^ ions removal were conducted over 0-30 min at an initial I^−^ ions concentration of 100 ppm and pH 2, with the solid-to-liquid ratio of 1 g L^−1^. At each time point, the solution was taken out and analyzed for the I^−^ ion using ICP-MS. The removal rate (*R*_%_) for I^−^ ions was calculated by the following equation:

| 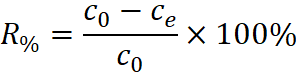 | (4) |
| --- | --- |

where *c*_0_ and *c*_e_ have the same meaning as Equation (1).

To investigate the effect of competing ions on the I^−^ removal performance of MIR-POP, SO_4_^2−^, NO_3_^−^, F^−^, Cl^−^, and Br^−^ were individually introduced into iodide solutions with an initial I^−^ ions concentration of 10 ppm. The molar ratios of competing ions to I^−^ ions were set at 50:1 and 100:1, respectively. The pH value of the mixed solution was adjusted to 2. A fixed amount of 10 mg MIR-POP was added into 10 mL of each prepared solution and stirred for 30 min. The residual concentrations of I^−^ ions in the supernatant were quantified using ICP-MS.

The recyclability of MIR-POP was evaluated through adsorption-desorption cycles. In a typical run, MIR-POP was dispersed in an aqueous solution containing 10 ppm I^−^ ions at the solid-to-liquid ratio of 1 g L^−1^ and subjected to light irradiation for 30 min to reach adsorption equilibrium. The adsorbent was then separated by filtration and the residual I^−^ ions concentration in the supernatant was quantified by ICP-MS. For desorption, the iodide-loaded MIR-POP was transferred into a 1 M NaCl solution and ultrasonicated for 2 h to release the adsorbed I^−^ ions. The regenerated material was washed thoroughly with deionized water and dried under vacuum at 60 °C. The recycled MIR-POP was subsequently reused in the next cycle. This adsorption-desorption process was repeated for five consecutive runs to assess the reusability of MIR-POP. To further assess potential material degradation during the repeated photooxidation, TOC analysis was performed on the supernatant after the fifth cycle. A 10 mL aliquot of the supernatant was mixed with 50 μL of 6 M HCl and ultrasonicated for 60 min to remove dissolved inorganic carbon. The treated solution was then analyzed using a TOC analyzer to quantify the residual organic carbon content.

To better approximate realistic wastewater environments, the I^−^ ions removal efficiency of MIR-POP was examined in a simulated mining effluent formulated according to reported leachate compositions from Chilean caliche deposits.^[1]^ The simulated wastewater was prepared to contain 10 ppm I^−^ ions, 130 ppm SO_4_^2−^ ions, 350 ppm Cl^−^ ions, 220 ppm Na^+^ ions, 20 ppm K^+^ ions, and 30 ppm Mg^2+^ ions, with the solution pH adjusted to 2. The different solid-to-liquid ratios of 0.1–1 g L^−1^ were examined using the prepared simulated mining wastewater and stirred continuously. Aliquots were collected at predetermined time intervals during the adsorption process for subsequent ICP-MS analysis.

Natural brine, as one of the most important reservoirs of iodine, was employed to evaluate the iodine extraction performance of MIR-POP. The raw brine collected from Dabuxun Lake in Qinghai Province, China, was first filtered to remove suspended solids, after which the pH was adjusted to 2, and the solution was used directly without further modification, thereby preserving its intrinsic ionic composition for adsorption tests. MIR-POP was immersed in filtered brine at the solid-to-liquid ratios of 1, 2, 5, 8, and 10 g L^−1^ followed by magnetic stirring. The residual concentration of I^−^ ions in the supernatant was analyzed using ICP-MS to evaluate the I^−^ ions extraction efficiency under realistic brine matrices.

Supporting Figures and Tables


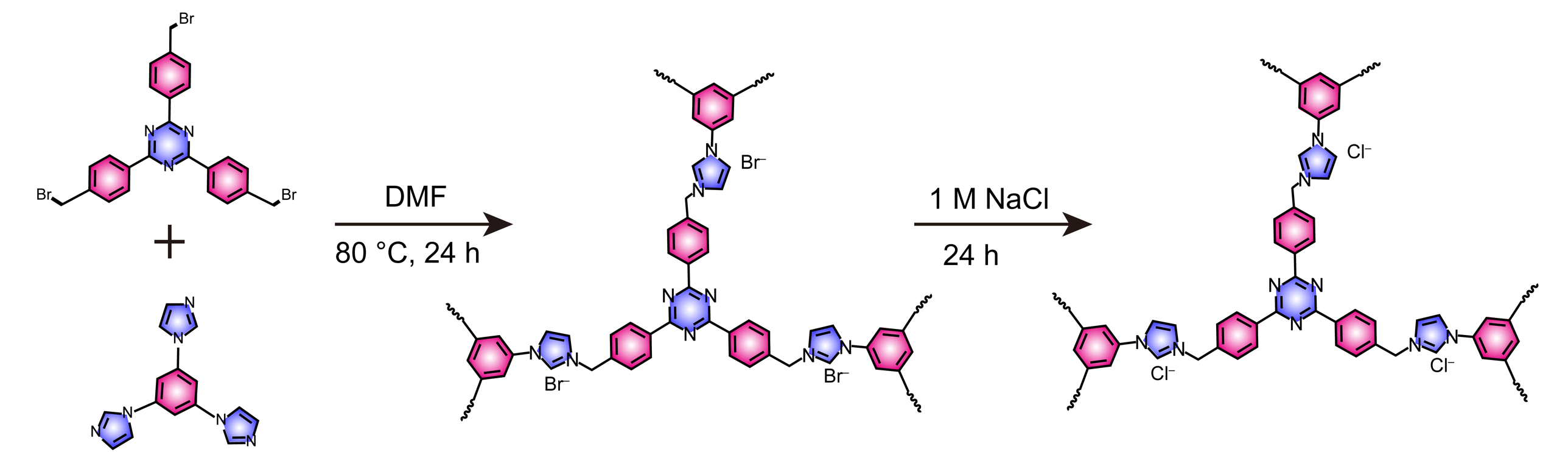


**Figure S1.** Synthesis route of MIR-POP.


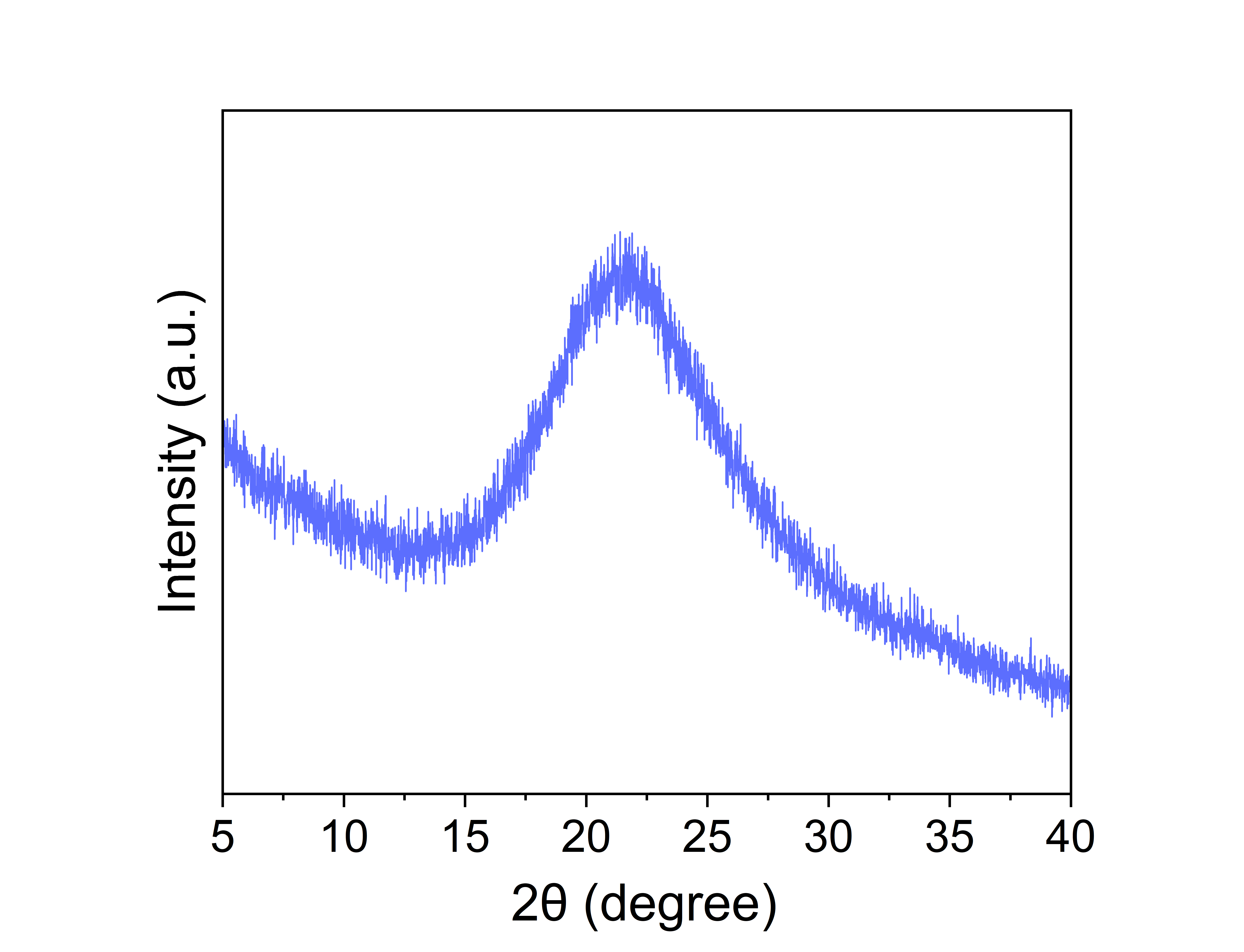


**Figure S2.** PXRD pattern of MIR-POP.


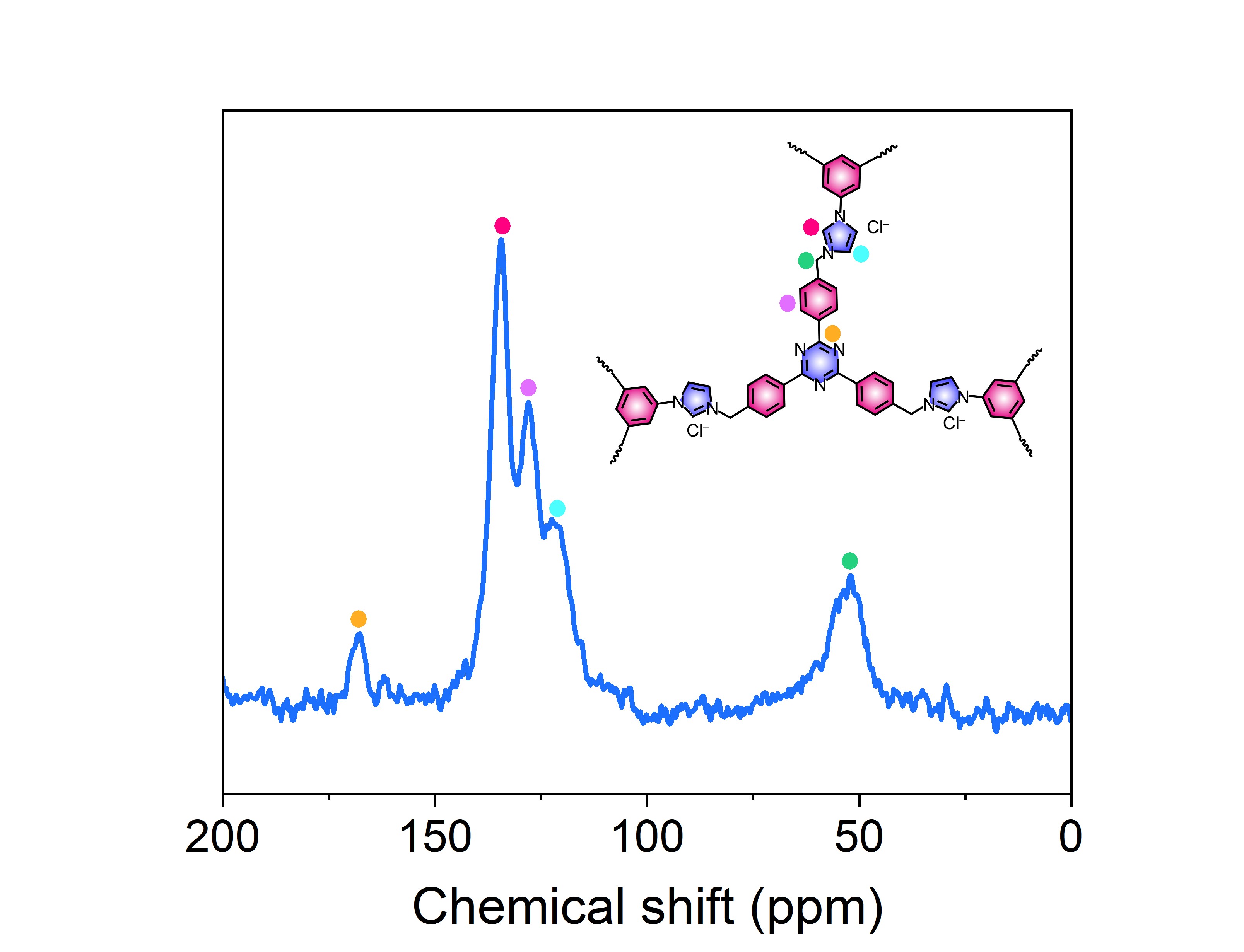


**Figure S3.** The solid-state ^13^C NMR spectrum of MIR-POP.


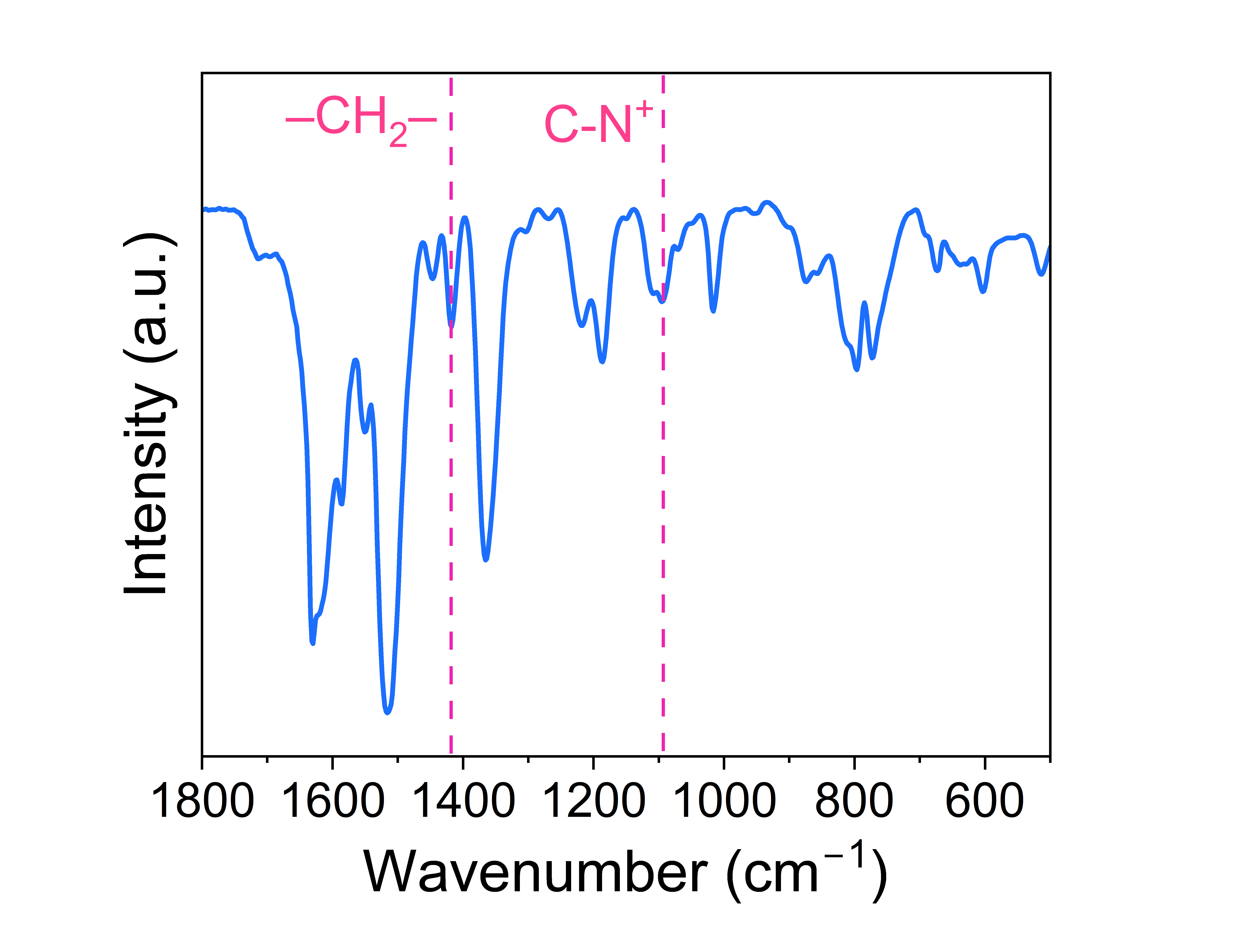


**Figure S4.** FT-IR spectrum of MIR-POP.


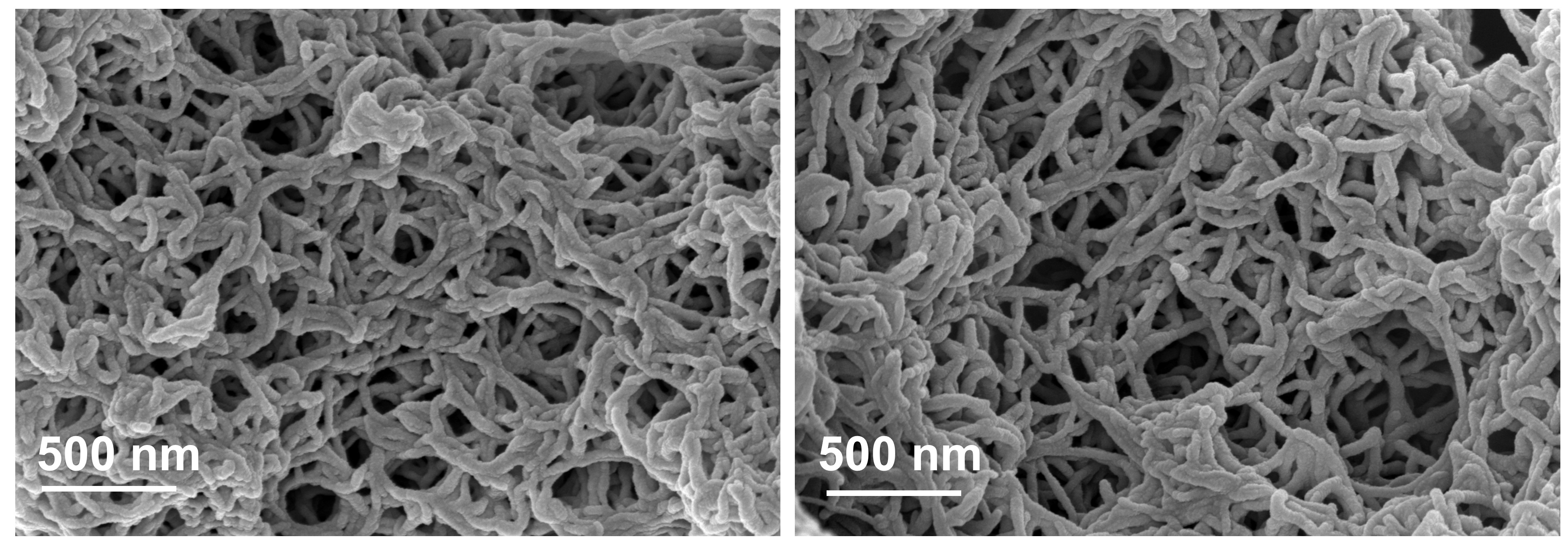


**Figure S5.** SEM images of MIR-POP.


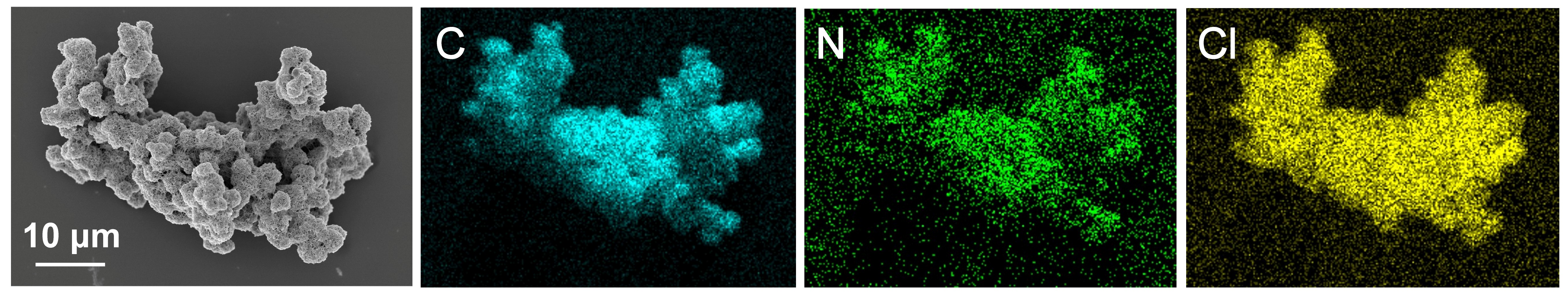


**Figure S6.** The element mapping of MIR-POP.


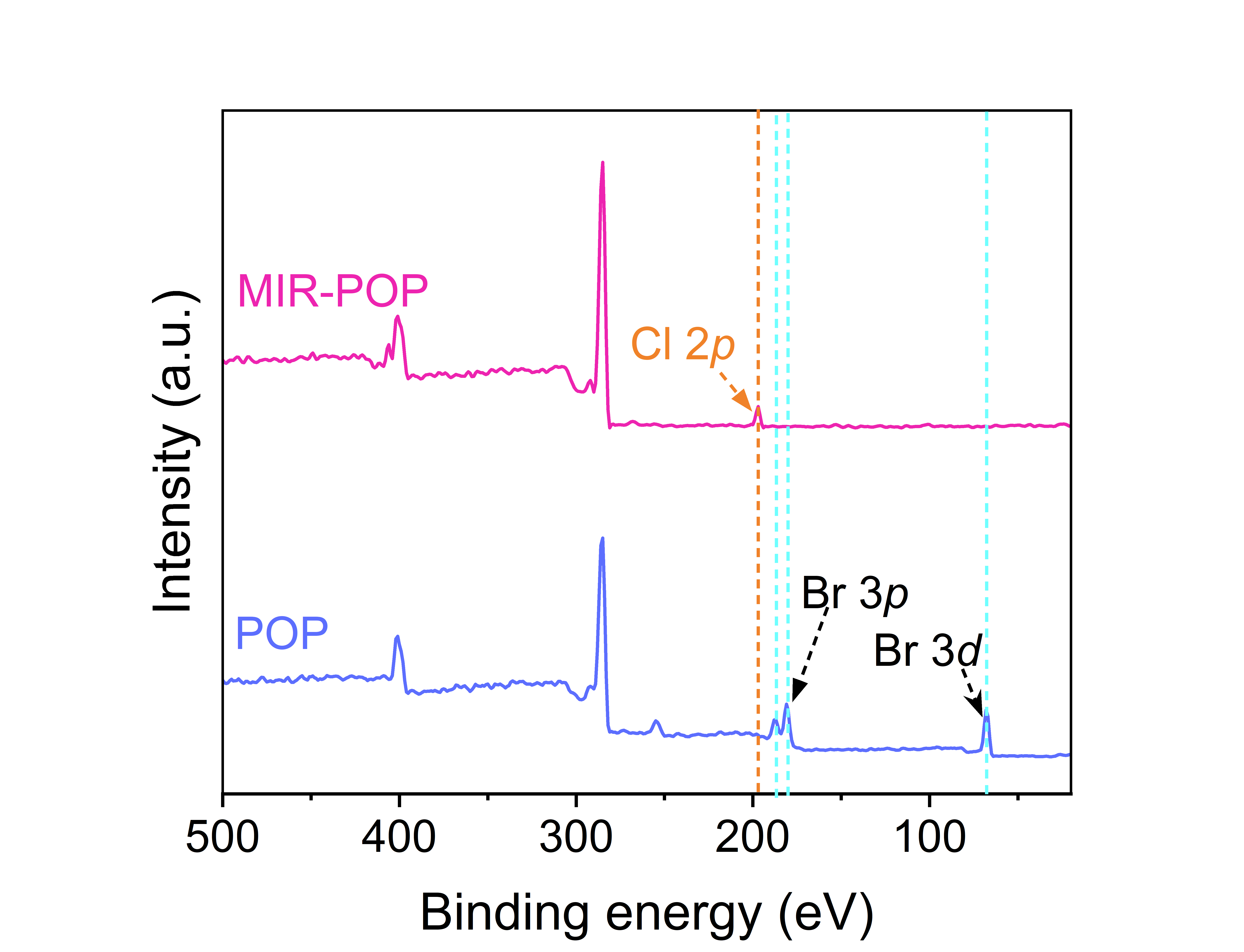


**Figure S7.** XPS spectra of pristine POP and MIR-POP.


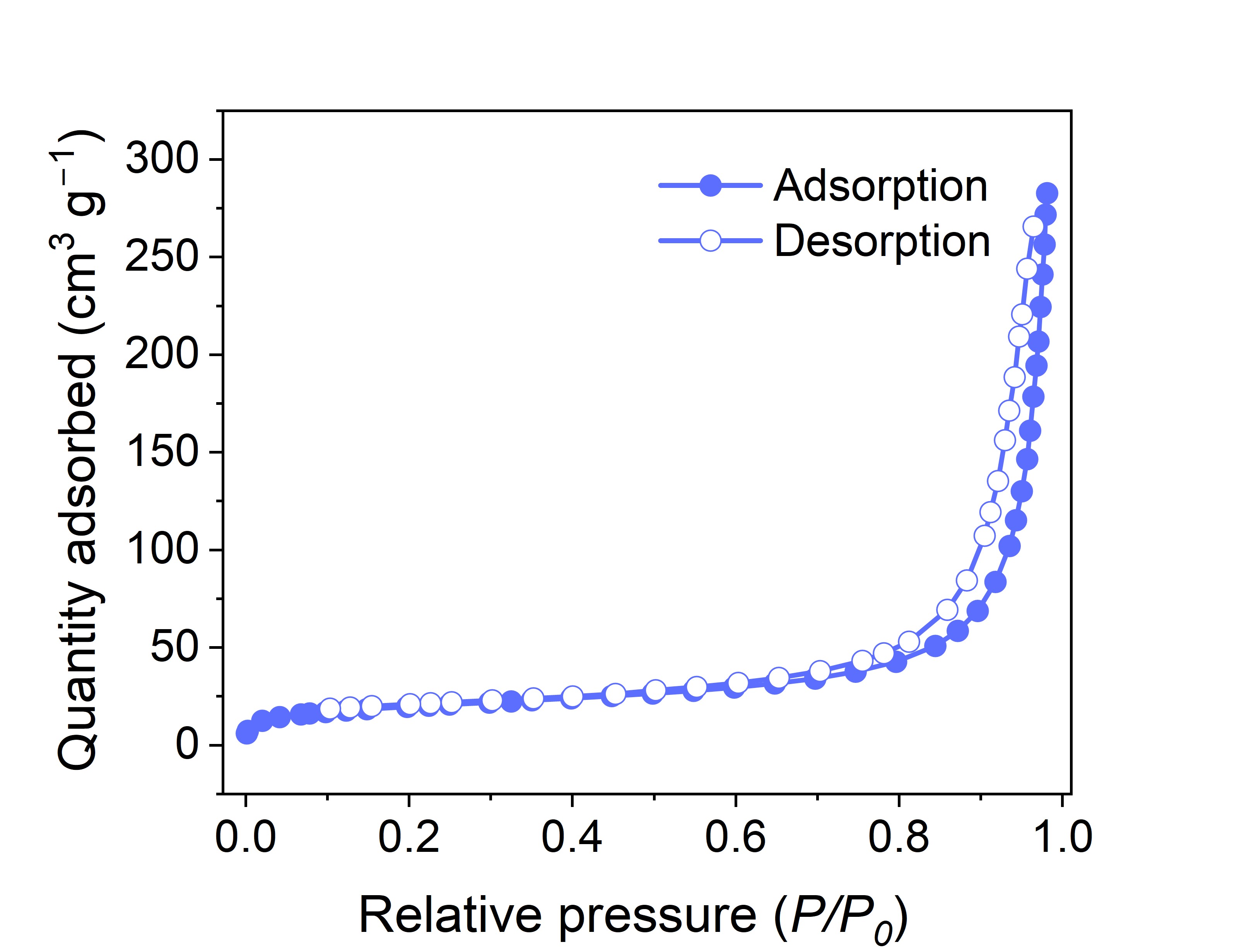


**Figure S8.** N_2_ adsorption-desorption isotherms of MIR-POP.


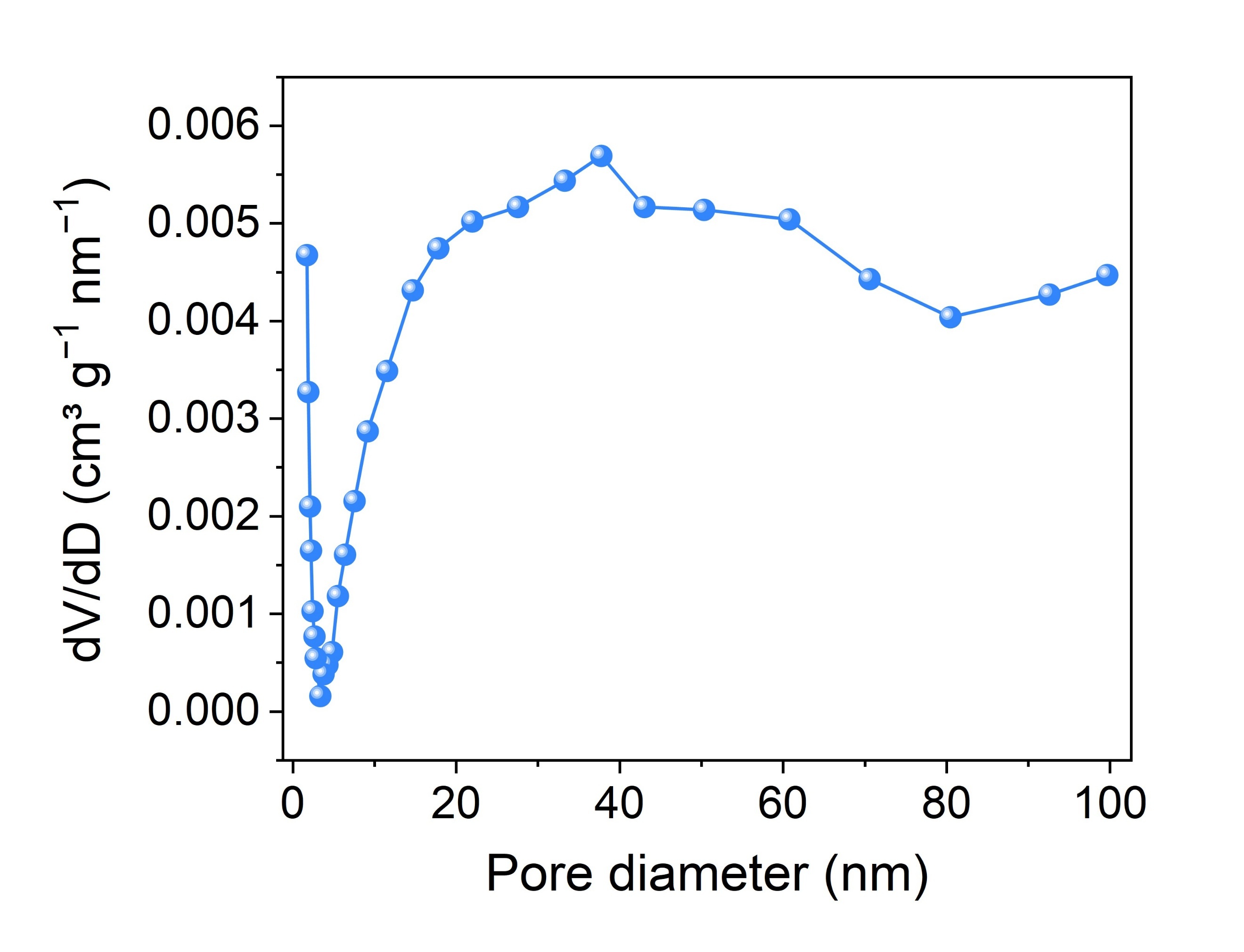


**Figure S9.** Pore size distribution of MIR-POP in the mesoporous region.


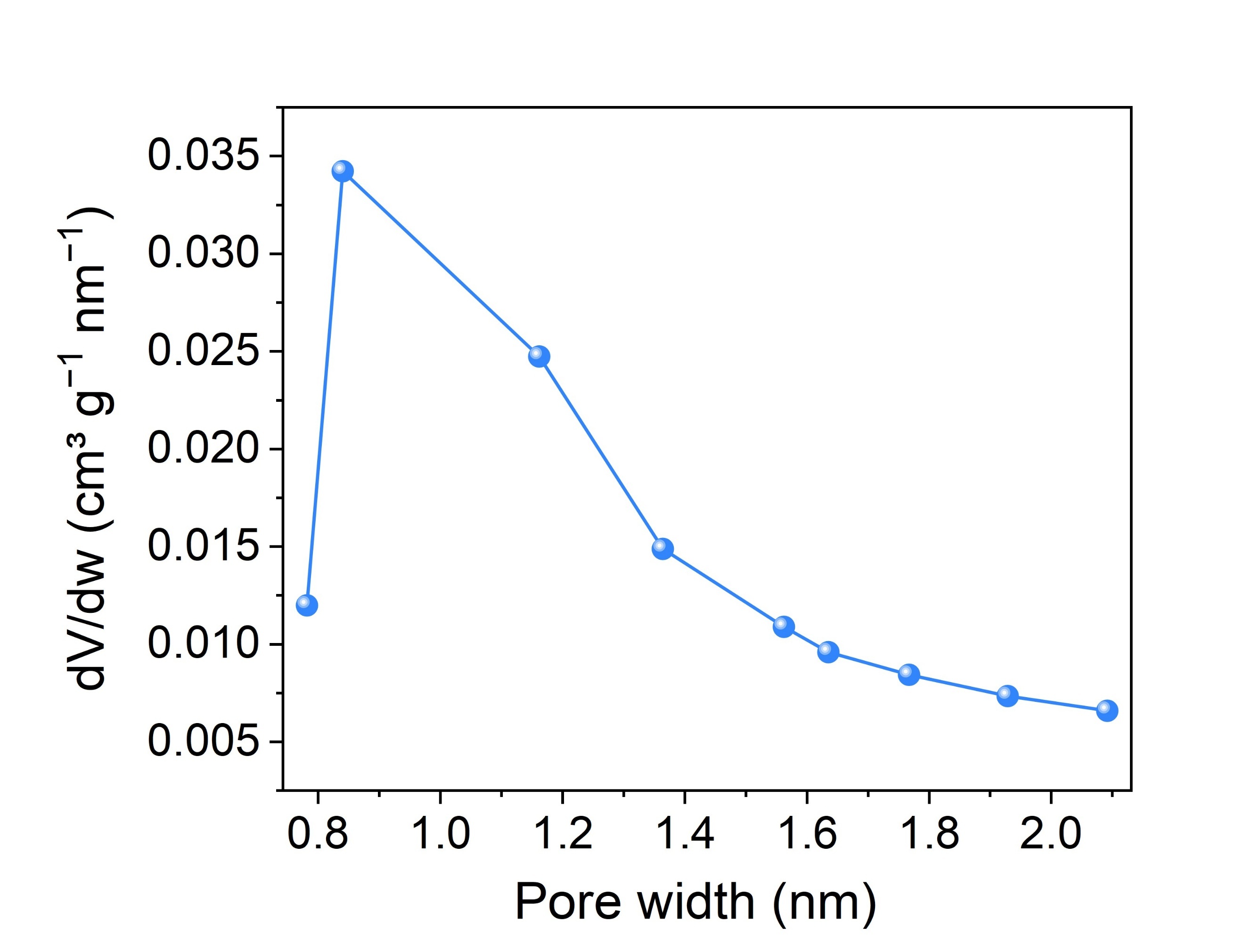


**Figure S10.** Pore size distribution of MIR-POP in the microporous region.


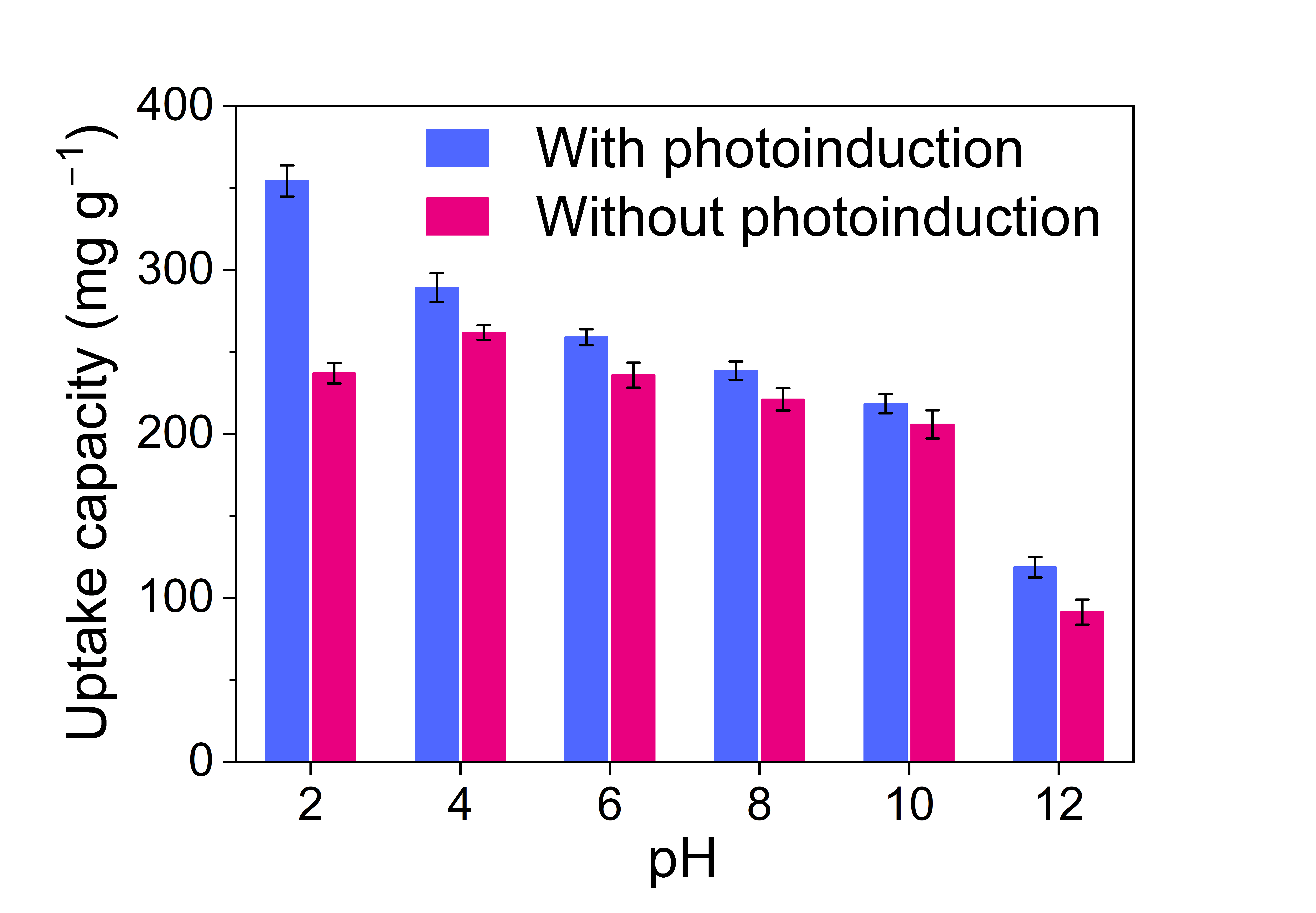


**Figure S11.** I^−^ ions uptake capacity of MIR-POP under different pH values with and without photoinduced conditions at an initial iodide concentration of 100 ppm (the concentration of MIR-POP is 0.05 mg mL^−1^).


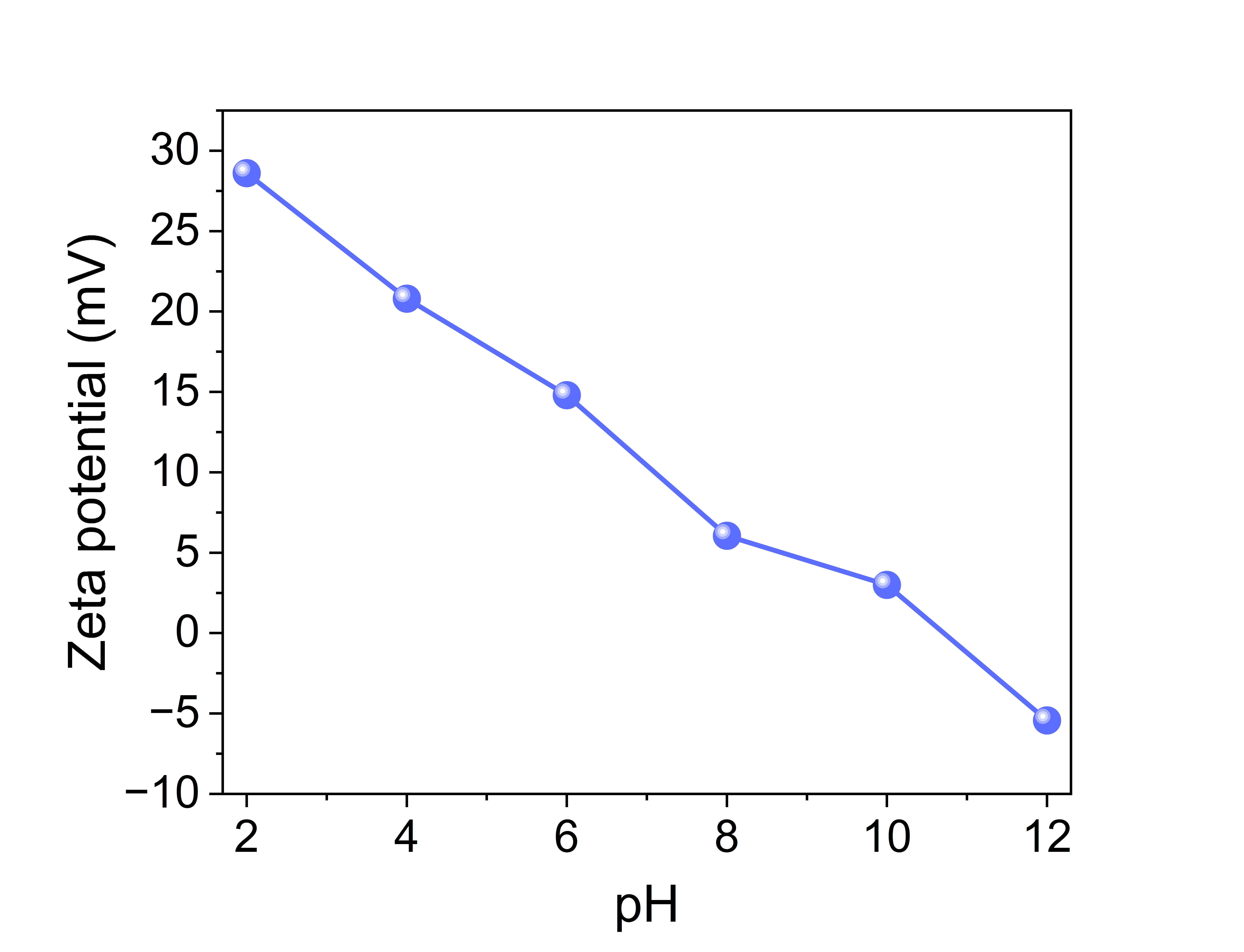


**Figure S12.** Zeta potential of MIR-POP at different pH values (the concentration of MIR-POP is 0.5 mg mL^−1^).


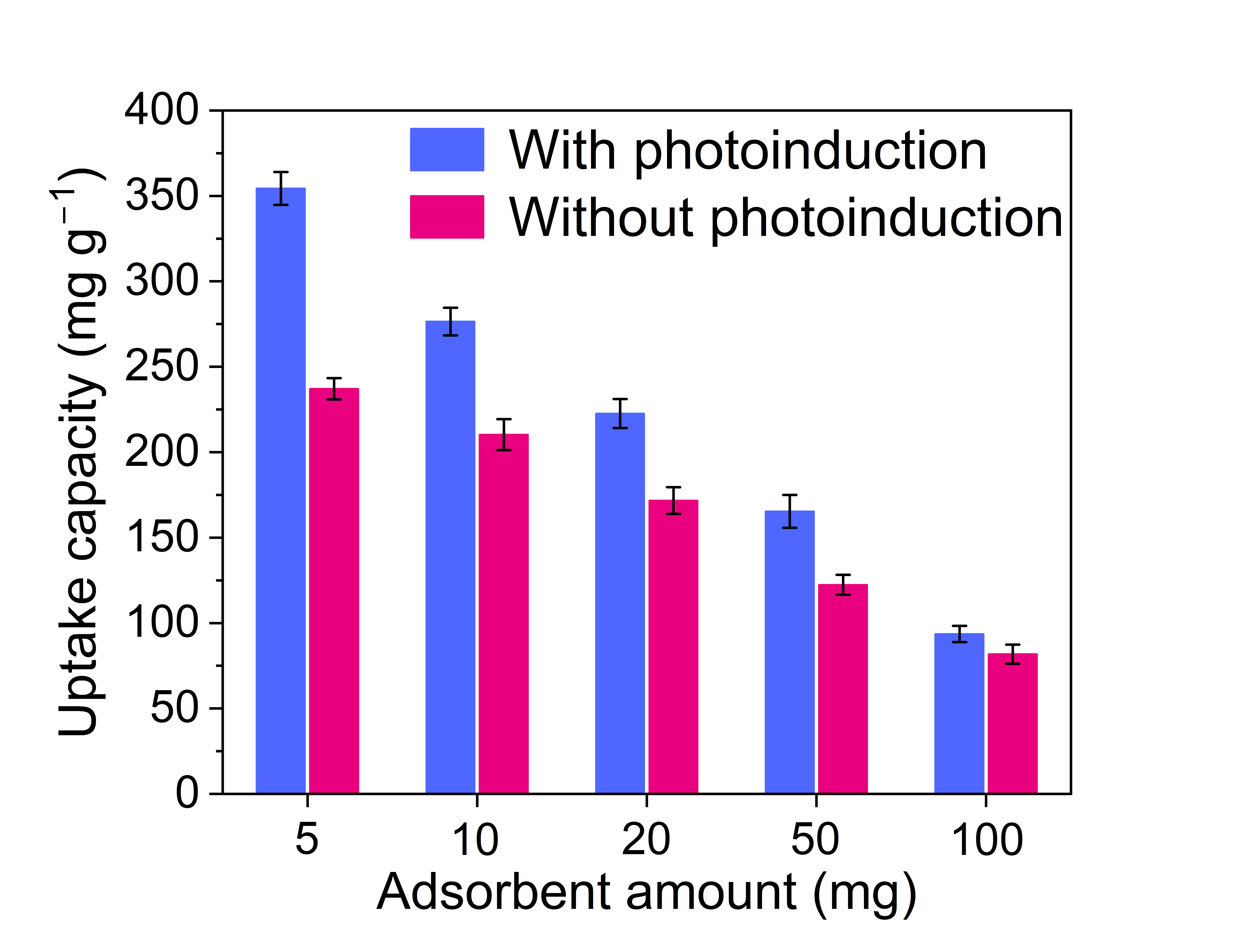


**Figure S13.** I^−^ ions uptake capacity of MIR-POP at different adsorbent amounts with and without photoinduced conditions at an initial iodide concentration of 100 ppm.


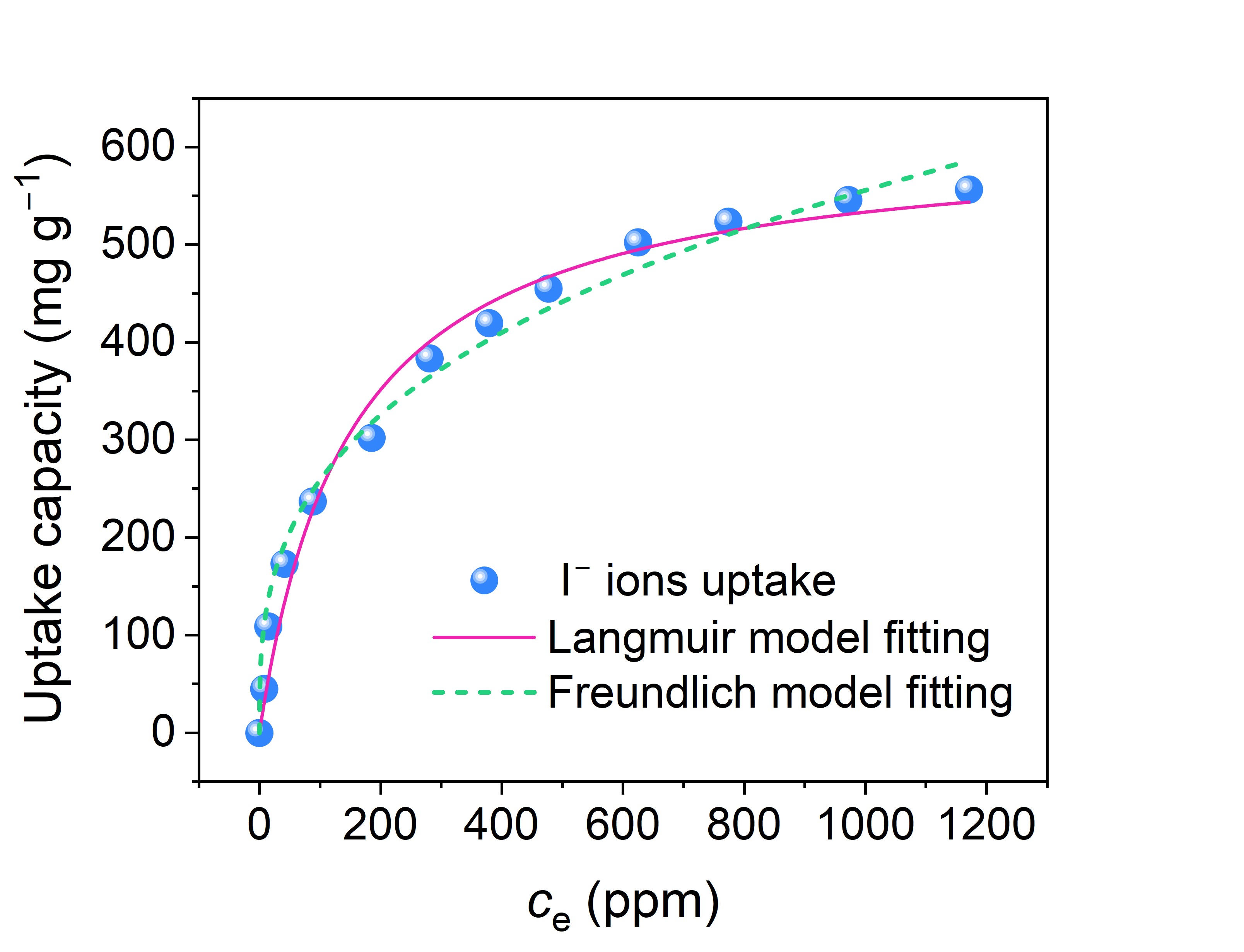


**Figure S14.** Uptake isotherms of MIR-POP for I^−^ ions at pH 2 without photoinduction.


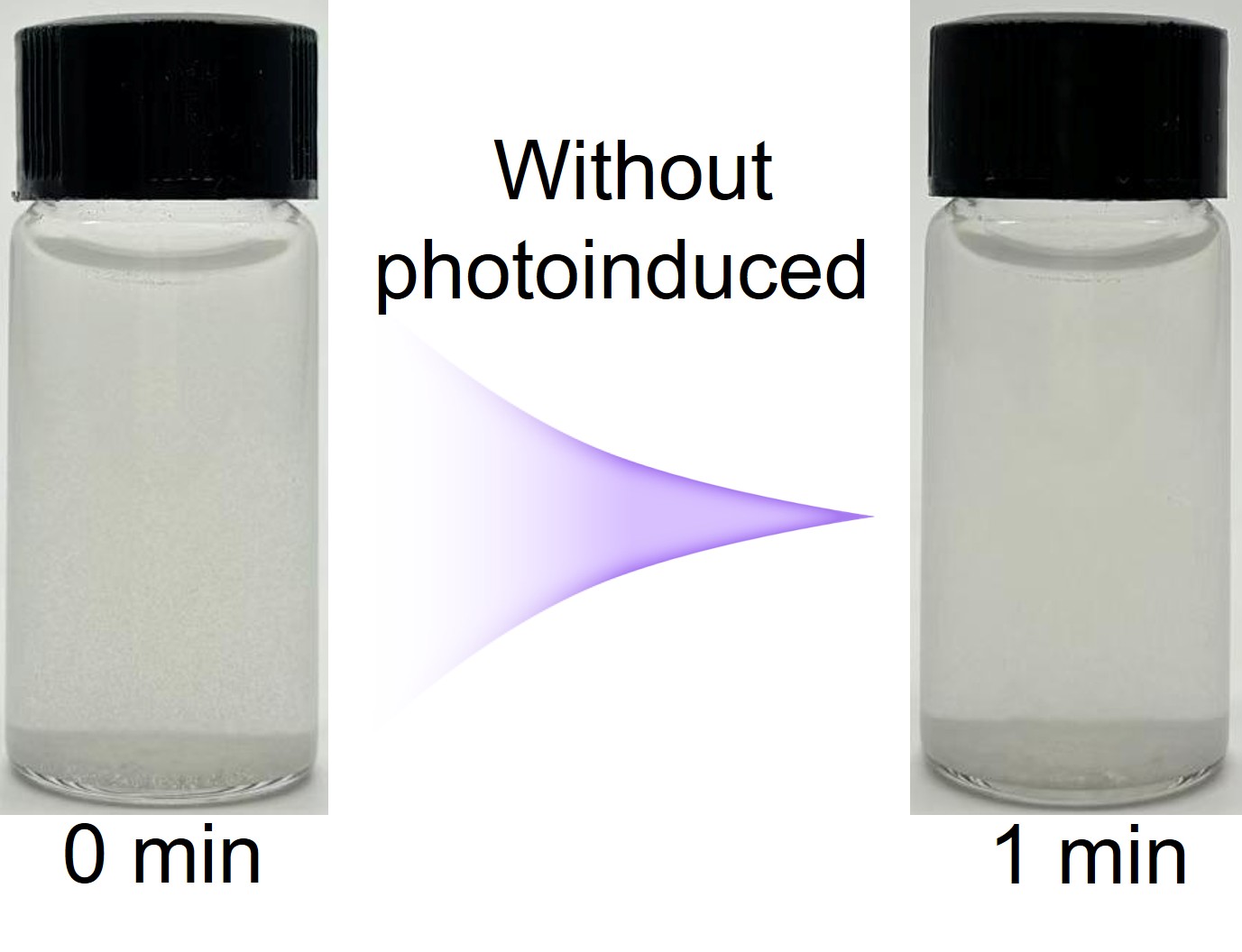


**Figure S15.** Color change of MIR-POP after 1 min exposure to 1200 ppm iodide solution without photoinduced conditions.


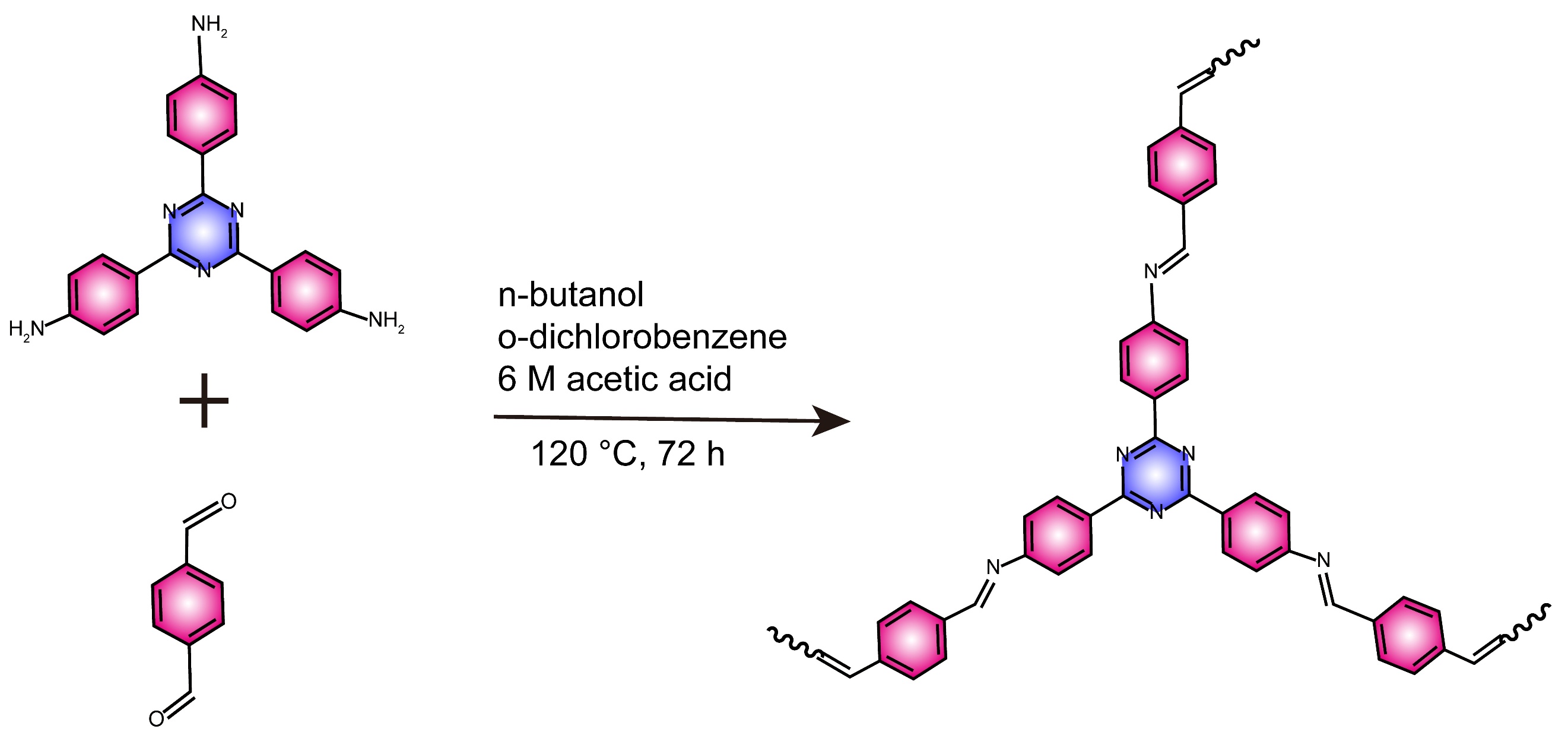


**Figure S16.** Synthesis route of non-MIR-POP.


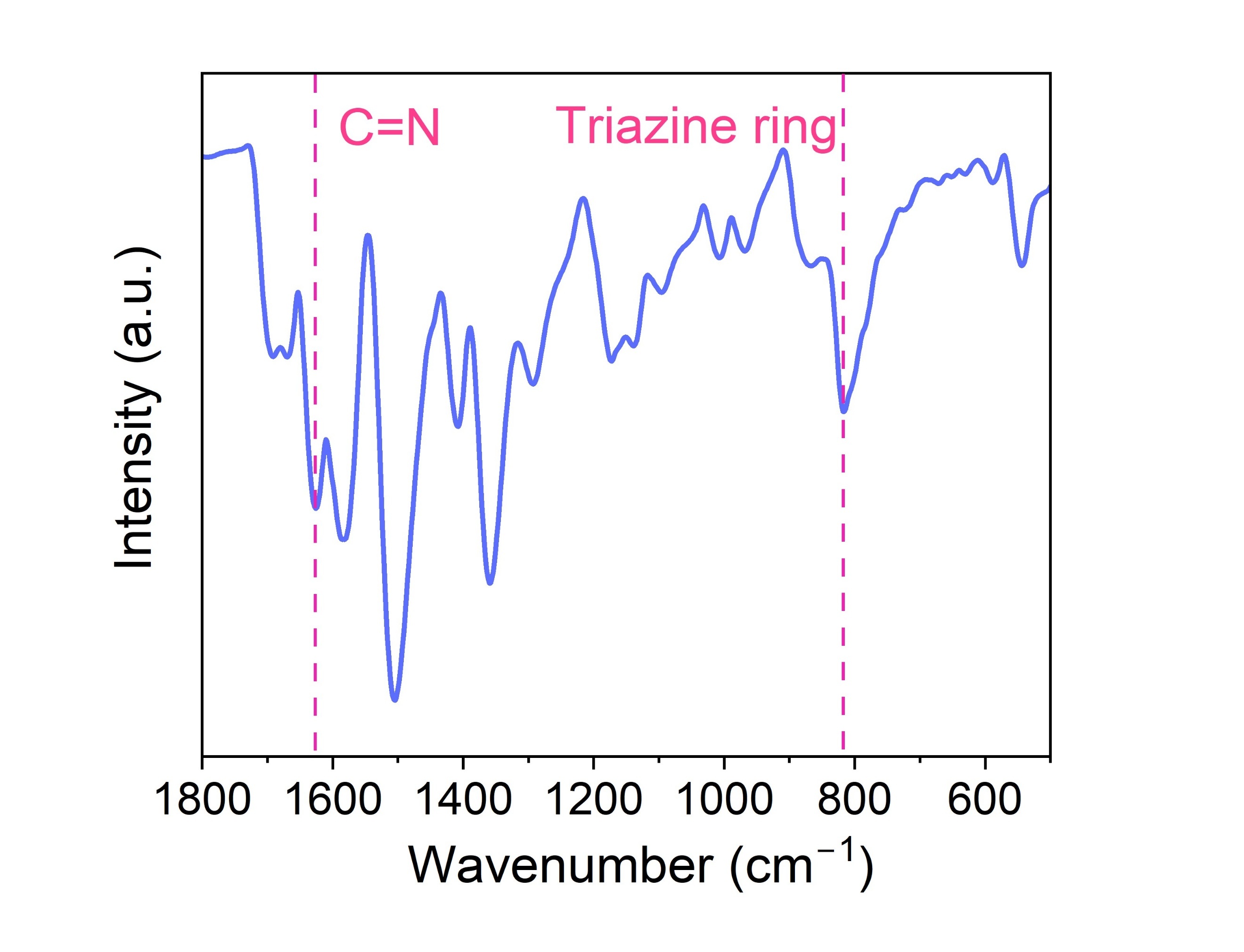


**Figure S17.** FT-IR spectrum of non-MIR-POP.


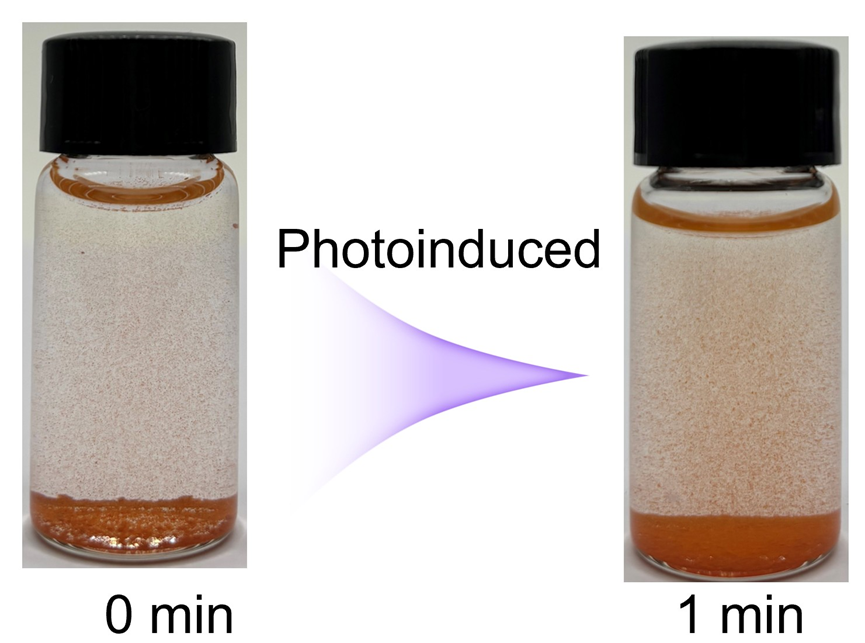


**Figure S18.** Color change of non-MIR-POP after 1 min exposure to 100 ppm iodide solution under photoinduced conditions.


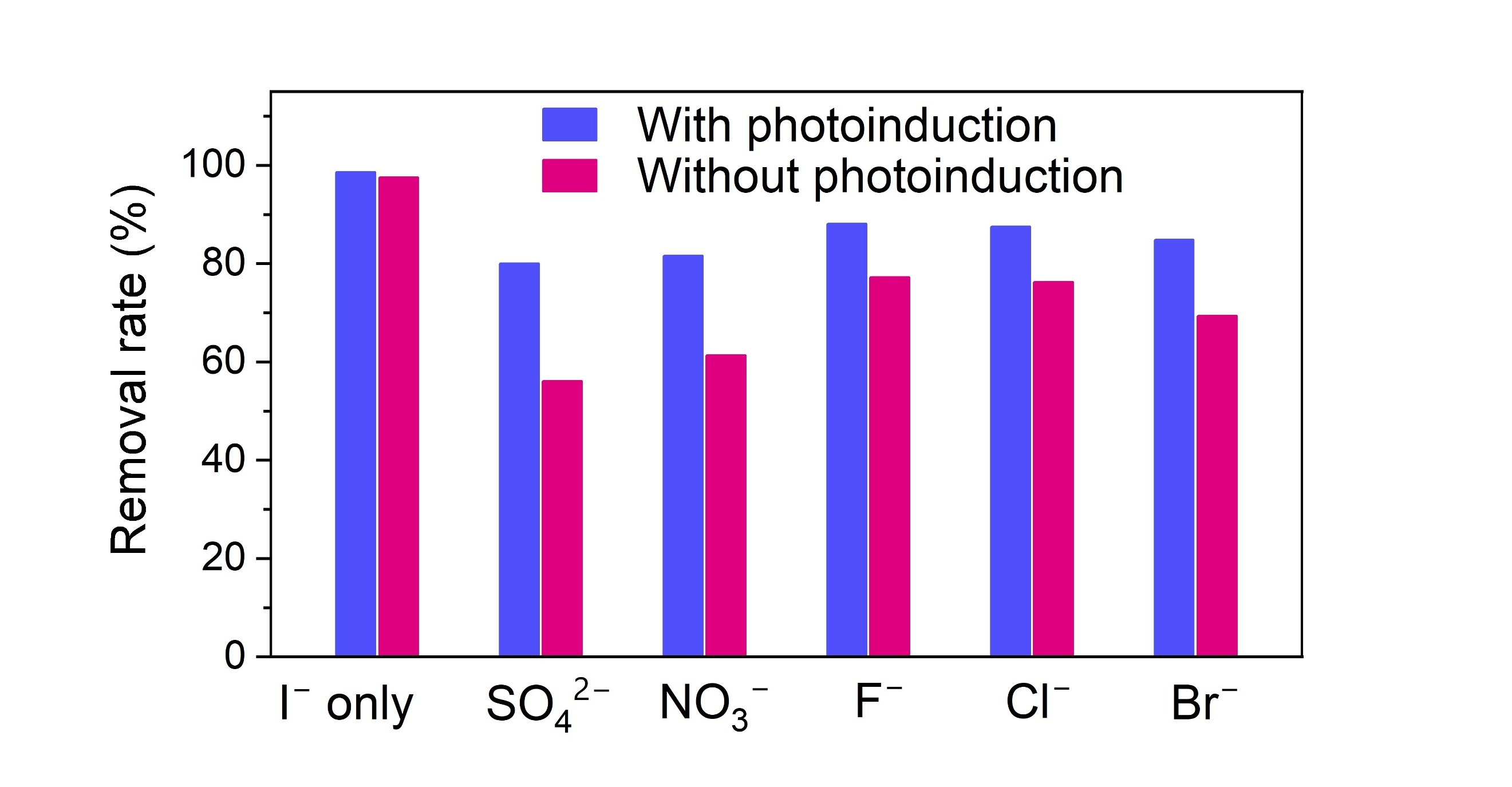


**Figure S19.** Effect of 100 equivalent competing anions on I^−^ ions removal from aqueous solutions.


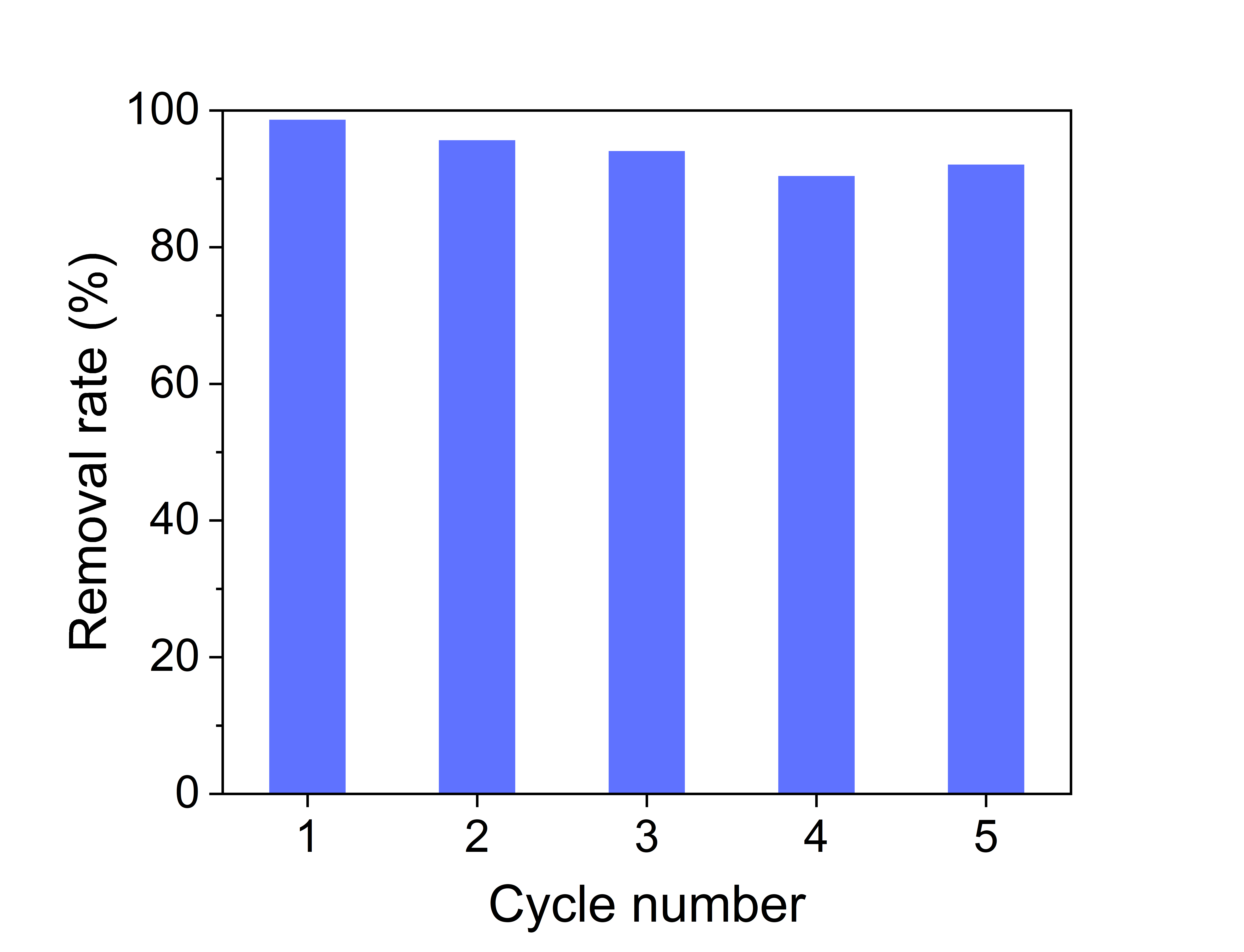


**Figure S20.** Reusability of MIR-POP for I^−^ ions removal.


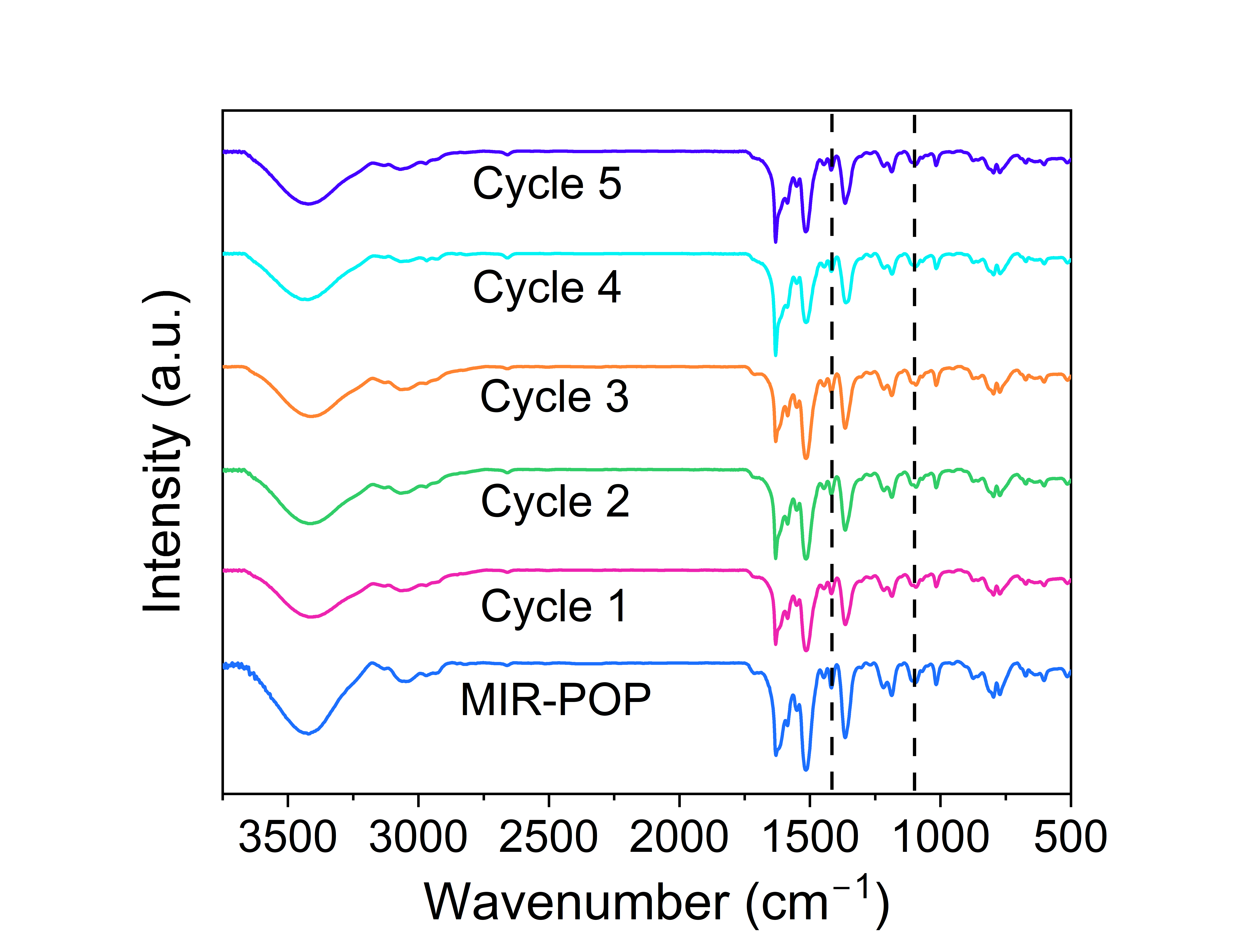


**Figure S21.** FT-IR spectra of MIR-POP before and after cycled.


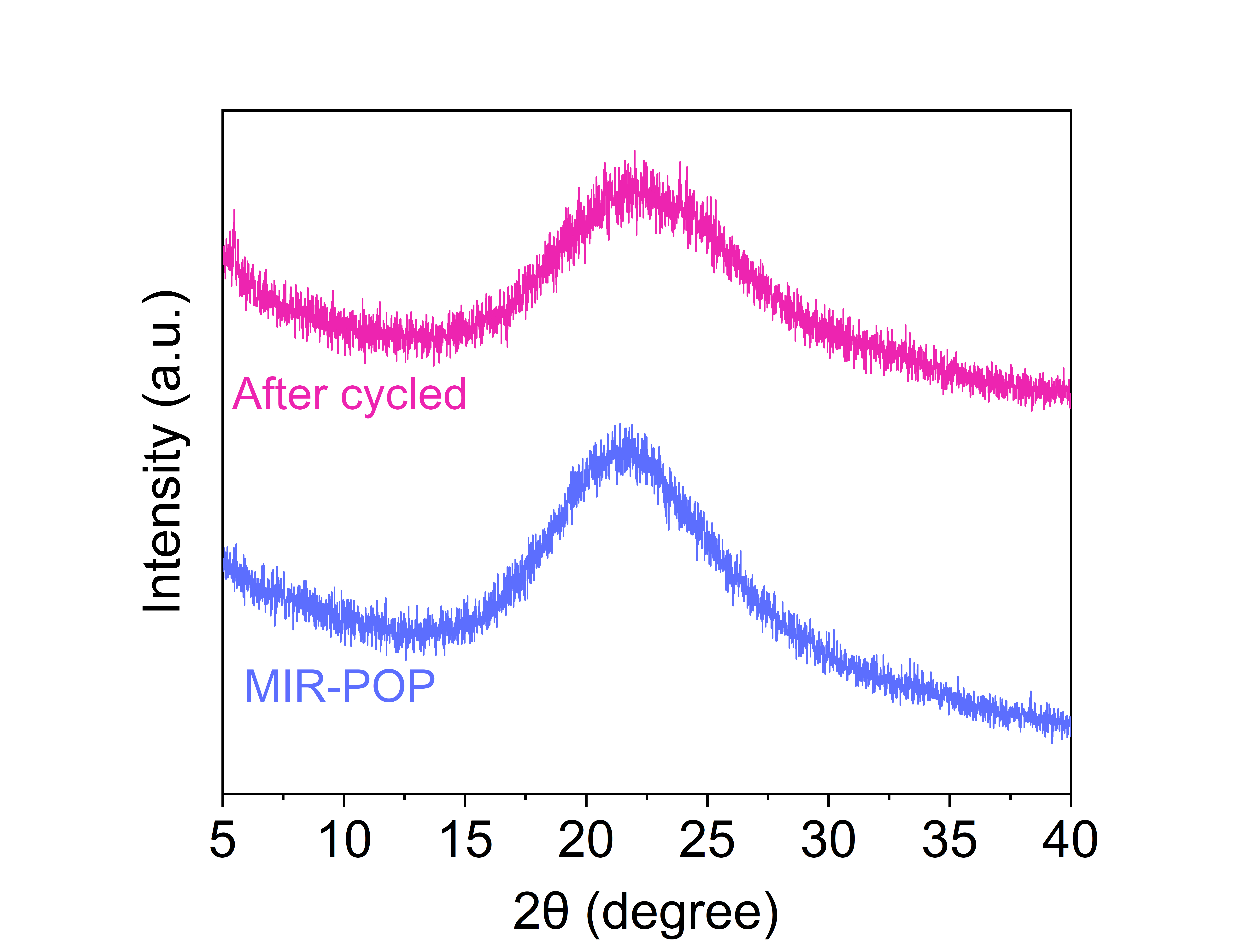


**Figure S22.** XRD patterns of MIR-POP before and after cycled.


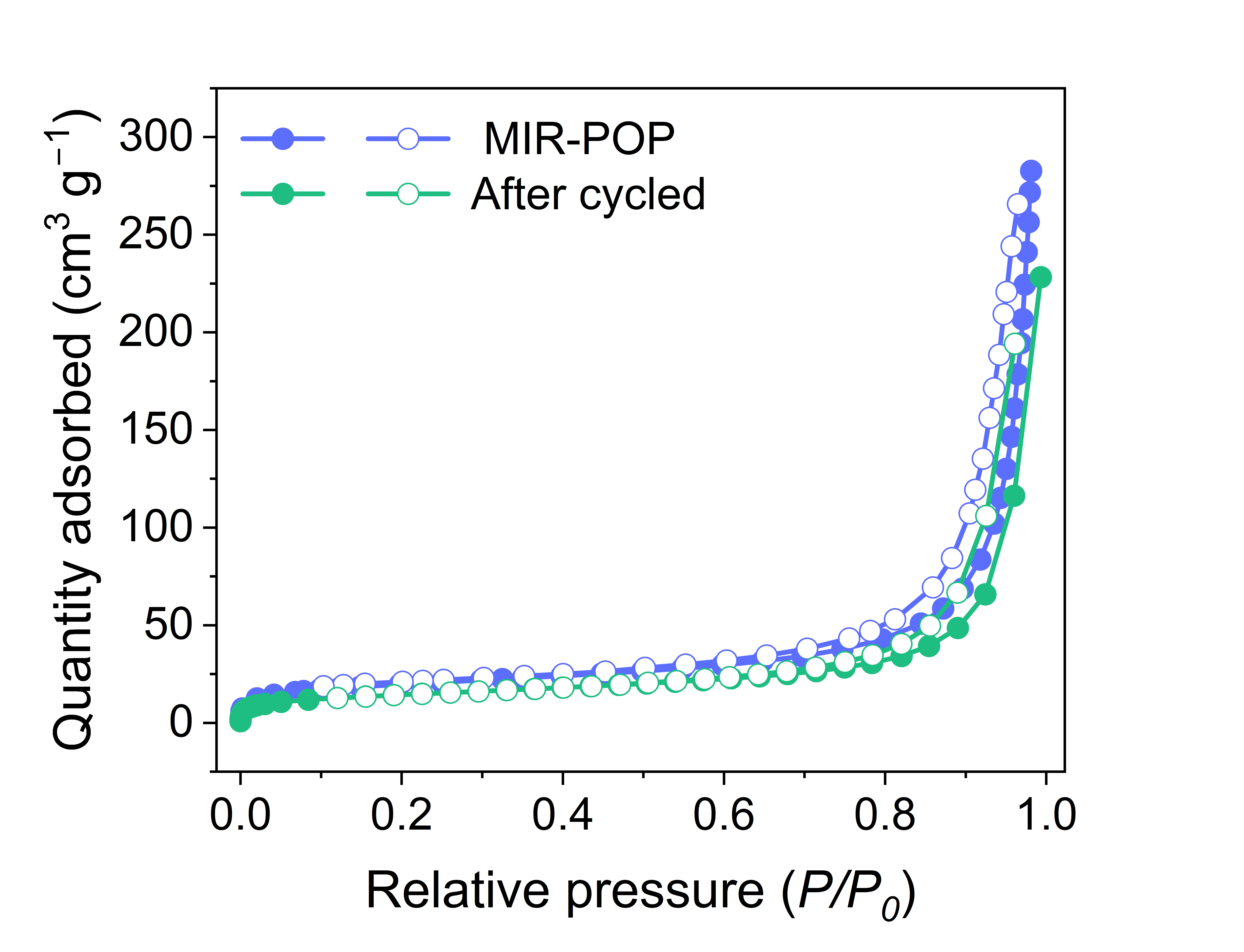


**Figure S23.** N_2_ adsorption-desorption isotherms of MIR-POP before and after cycled.


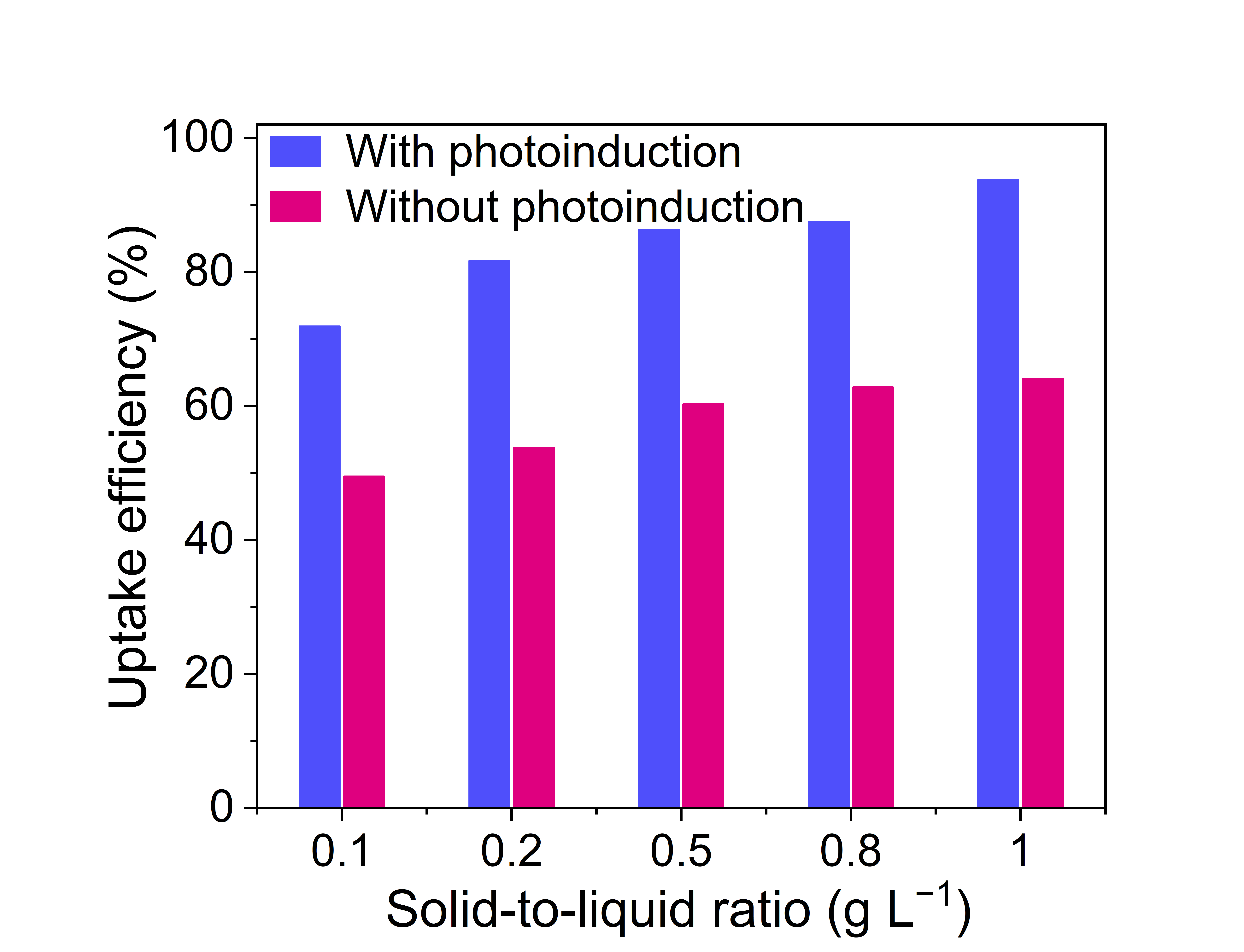


**Figure S24.** Uptake efficiency of I^−^ ions in simulated mining wastewater at different solid-to-liquid ratios.


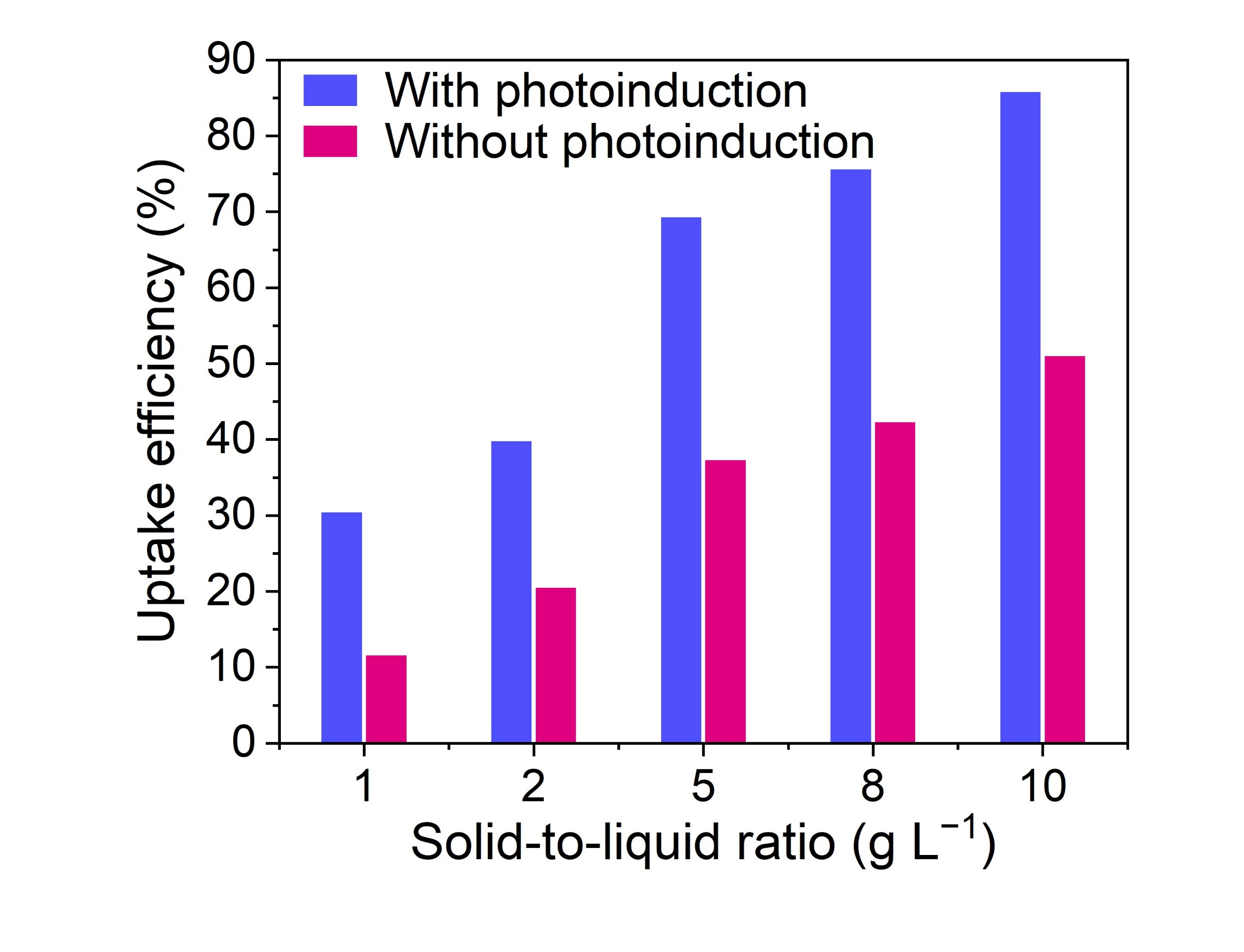


**Figure S25.** Uptake efficiency of I^−^ ions in natural brine at different solid-to-liquid ratios.


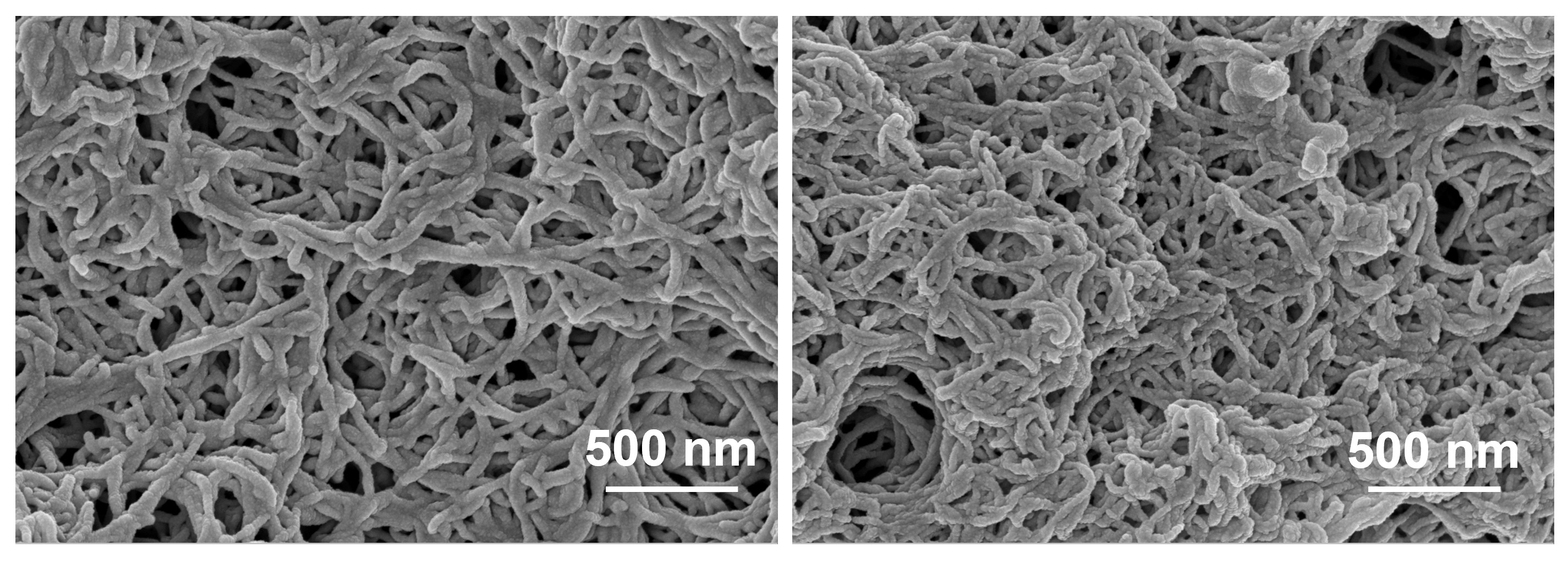


**Figure S26.** SEM images of MIR-POP@I^−^ obtained under light irradiation at pH 2.

**Table S1.** The fitting results of the adsorption isotherm fitted by the Langmuir and the Freundlich model for aqueous I^−^ ions adsorption on MIR-POP without photoinduction.

|  | Langmuir | | | Freundlich | | |
| --- | --- | --- | --- | --- | --- | --- |
|  | *q*_mL_  (mg g^−1^) | *K*_L_  (L mg ^−1^) | *R*^2^ | *K*_F_  ((mg g^−1^) (L mg^−1^)^1/n^) | 1/*n*_F_ | *R*^2^ |
| Without photoinduction | 612.62 | 0.01 | 0.9821 | 556.14 | 0.33 | 0.9314 |

**Table S2.** Comparison of MIR-POP with representative adsorbents reported in previous studies in terms of I⁻ ions adsorption capacity.

| Adsorbents | Adsorption capacity (mg g^−1^) | Reference |
| --- | --- | --- |
| MIR-POP | 853.06 | This study |
| Ag-MSHC-6 | 771.6 | [2] |
| Ag^0^-UiO-66-(OH)_2_ | 531.98 | [3] |
| SOF-HTNI | 503.35 | [4] |
| COF-V | 437.8 | [5] |
| Cu/Al_2_O_3_ aerogel | 407.6 | [6] |
| N-AF | 347 | [7] |
| Fe_3_O_4_@SiO_2_-MPS-PILs | 335.8 | [8] |
| calcined MgFe | 317.5 | [9] |
| Ag_2_O–SNF | 293.46 | [10] |
| NiAl LDH | 266.7 | [11] |
| Ag_4/3_@Zn_3_Al_1_-LDH | 256 | [12] |
| 5%Ag@Cu–C | 247.1 | [13] |
| MIL-101(Cr)-SO_3_Ag | 244.2 | [14] |
| Cu2O@Cu/Al-CLDH | 240.7 | [15] |
| Ag^+^@UiO-66-(COOH)_2_ | 235.5 | [16] |
| iPAF-TEPT | 220 | [17] |
| CoAl LDH | 212.09 | [11] |
| 15%-Bi@MIL | 189.6 | [18] |
| NPOP-TIPA | 180 | [19] |

# References

[1] J. I. Ordonez, L. Moreno, M. E. Mellado, L. A. Cisternas, *Int. J. Miner. Process.* **2014**, *126*, 10-17.

[2] H. L. Li, Y. Li, B. L. Li, D. B. Liu, Y. Z. Zhou, *Chemosphere* **2020**, *252*, 126448.

[3] T. Wang, H. F. Zhao, X. D. Zhao, D. H. Liu, *J. Solid State Chem.* **2022**, *305*, 122680.

[4] L. Feng, J. Zhang, J. Zhang, X. Cao, Z. Guo, Y. Yuan, N. Wang, *Research* **2025**, *8*, 0608.

[5] X. H. Tian, G. W. Zhou, J. W. Xi, R. F. Sun, X. F. Zhang, G. Wang, L. Mei, C. Hou, L. P. Jiang, J. H. Qiu, *Sep. Purif. Technol.* **2023**, *310*, 123160.

[6] X. Y. Zhou, P. Mao, H. R. Jin, W. X. Huang, A. T. Gu, K. W. Chen, S. Yun, J. Chen, Y. Yang, *J. Hazard. Mater.* **2023**, *443*, 130349.

[7] Z. Liao, N. Pan, J. Liu, C. Ma, X. Xia, J. Deng, G. Yang, X. Li, Z. Chen, W. Cheng, W. Zhang, X. Nie, F. Dong, *J. Environ. Chem. Eng.* **2024**, *12*, 111783.

[8] X. Li, Y. Shen, F. Jin, J. Zhang, Y. Yang, X. Qu, *Sep. Purif. Technol.* **2024**, *335*, 126210.

[9] J. Kang, T. G. Levitskaia, S. Park, J. Kim, T. Varga, W. Um, *Chem. Eng. J.* **2020**, *380*, 122408.

[10] W. J. Mu, Q. H. Yu, X. L. Li, H. Y. Wei, Y. Jian, *RSC Adv.* **2016**, *6*, 81719-81725.

[11] J. Kang, F. Cintron-Colon, H. Kim, J. Kim, T. Varga, Y. G. Du, O. Qafoku, W. Um, T. G. Levitskaia, *Chem. Eng. J.* **2022**, *430*, 132788.

[12] X. Yuan, W. Yu, X. Xiao, L. Wang, Q. Wan, *J. Solid State Chem.* **2024**, *335*, 124731.

[13] C. H. Gong, Z. Y. Li, K. W. Chen, A. T. Gu, P. Wang, Y. Yang, *J. Environ. Radioact.* **2023**, *265*, 107211.

[14] X. D. Zhao, X. Han, Z. J. Li, H. L. Huang, D. H. Liu, C. L. Zhong, *Appl. Surf. Sci.* **2015**, *351*, 760-764.

[15] C. H. Gong, Z. Y. Li, K. W. Chen, A. T. Gu, P. Wang, Y. Yang, *J. Radioanal. Nucl. Chem.* **2023**, *332*, 2793-2805.

[16] J. Zhang, S. L. Yang, L. Shao, Y. M. Ren, J. L. Jiang, H. S. Wang, H. Tang, H. Deng, T. F. Xia, *Molecules* **2022**, *27*, 8547.

[17] Y. Ma, J. Pan, H. Rong, Y. Zhang, L. Liu, Y. Guo, J. Ai, Y. Yuan, N. Wang, *Adv. Sci.* **2025**, *12*, 2500993.

[18] W. J. Xu, W. S. Zhang, J. X. Kang, B. J. Li, *J. Solid State Chem.* **2019**, *269*, 558-565.

[19] J. Pan, Y. Ma, H. Rong, Y. Zhang, Y. Guo, C. Gan, L. Liu, Y. Yuan, N. Wang, *Sep. Purif. Technol.* **2025**, *368*, 133025.
